# An improved chronology for the Middle Stone Age at El Mnasra cave, Morocco

**DOI:** 10.1371/journal.pone.0261282

**Published:** 2022-02-11

**Authors:** Eslem Ben Arous, Anne Philippe, Qingfeng Shao, Daniel Richter, Arnaud Lenoble, Norbert Mercier, Maïlys Richard, Emmanuelle Stoetzel, Olivier Tombret, Mohamed Abdeljalil El Hajraoui, Roland Nespoulet, Christophe Falguères

**Affiliations:** 1 Pan-African Evolution Research Group, Max Planck Institute for the Science of Human Evolution, Jena, Germany; 2 Geochronology and Geology Programme, Centro Nacional de Investigación sobre la Evolución Humana (CENIEH), Burgos, Spain; 3 Histoire Naturelle de l’Homme Préhistorique (HNHP, UMR 7194)—Muséum National d’Histoire Naturelle, Sorbonne Universités, CNRS, UPVD, Paris, France; 4 Laboratoire de Mathématiques Jean Leray, Université de Nantes, Nantes, France; 5 Key Laboratory of Virtual Geographic Environment (Nanjing Normal University), Ministry of Education, Nanjing, China; 6 Department of Human Evolution, Max Planck Institute for Evolutionary Anthropology, Leipzig, Germany; 7 De la Préhistoire à l’Actuel: Culture, Environnement et Anthropologie, CNRS, Université Bordeaux, MCC, UMR 5199 PACEA, Pessac, France; 8 Institut de Recherche sur les Archéomatériaux, UMR 5060 CNRS, Université Bordeaux Montaigne, Centre de Recherche en Physique Appliquée à l’Archéologie (CRP2A), Maison de l’Archéologie, Pessac, France; 9 Abteilung für Ältere Urgeschichte und Quartärökologie, Institut für Ur- und Frühgeschichte und Archäologie des Mittelalters, University of Tübingen, Schloss Hohentübingen, Tübingen, Germany; 10 Archéozoologie, Archéobotanique: Sociétés, Pratiques et Environnements (AASPE, UMR 7209), Muséum National d’Histoire Naturelle, Sorbonne Universités, CNRS, Paris, France; 11 Institut National des Sciences de l’Archéologie et du Patrimoine (INSAP), Rabat, Morocco; Universita degli Studi di Milano, ITALY

## Abstract

North African coastal Middle Stone Age (MSA) sites are key to study the development and expansion of early *H*. *sapiens*. El Mnasra cave on the Atlantic coast of Morocco (Témara region) is a crucial site associated with MSA archaeological materials considered advanced cognitive hallmarks of behavioural innovation, such as numerous Nassariidae perforated shells, hematite pigments, bones industry and coastal resources exploitation. We provide new trapped-charges dates (OSL and combined US-ESR ages). Our Bayesian modelling strengthens the new lithostratigraphic interpretation of the cave stratigraphic units (US) and we propose an updated chronostratigraphic model for the Middle Stone Age archaeo-sequence of El Mnasra Cave. We confirm a human presence between 124–104 ka, earlier than what the previous OSL and US-ESR data showed. Our time range intervals allowed us to also extend the age of the MSA occupations considerably to the MIS 4/3 (~62–30 ka), marked by the disappearance of the Nassariidae perforated shells. Outstandingly, our model pushed back the age of the largest record of Nassariidae perforated shells and placed the age of their use by the Aterian groups at El Mnasra from the MIS 5d-5b (~115–94 ka).

## Introduction

Over the last twenty years, northern, southern and eastern African sites have yielded Middle Stone Age (MSA) archaeological materials often hailed as advanced symbolic and cognitive hallmarks of so-called behavioural innovations. In these regions, these artefacts (e.g. bifacial foliated stone tools, ochre and pigments, perforated marine shell beads, bone artefacts, engraved stones/ochre and ostrich eggshells) are between ~150 and ~50 thousand years (ka) old and associated with *Homo sapiens* MSA occupations [1–7]. Some of the sites that delivered these artefacts are coastal sites associated with evidence of marine resources exploitation, notably through the presence of marine shells [4, 8–10]. It has been suggested that such a diet may have contributed to the emergence of cognitive and behavioural innovations in ancient populations because the fatty acids provided by this sort of seafood can boost brain development [11]. Coastal areas may also have facilitated human dispersal because these regions have always presented a milder climate and a certain richness in food resources [12, 13].

**Fig 1:**
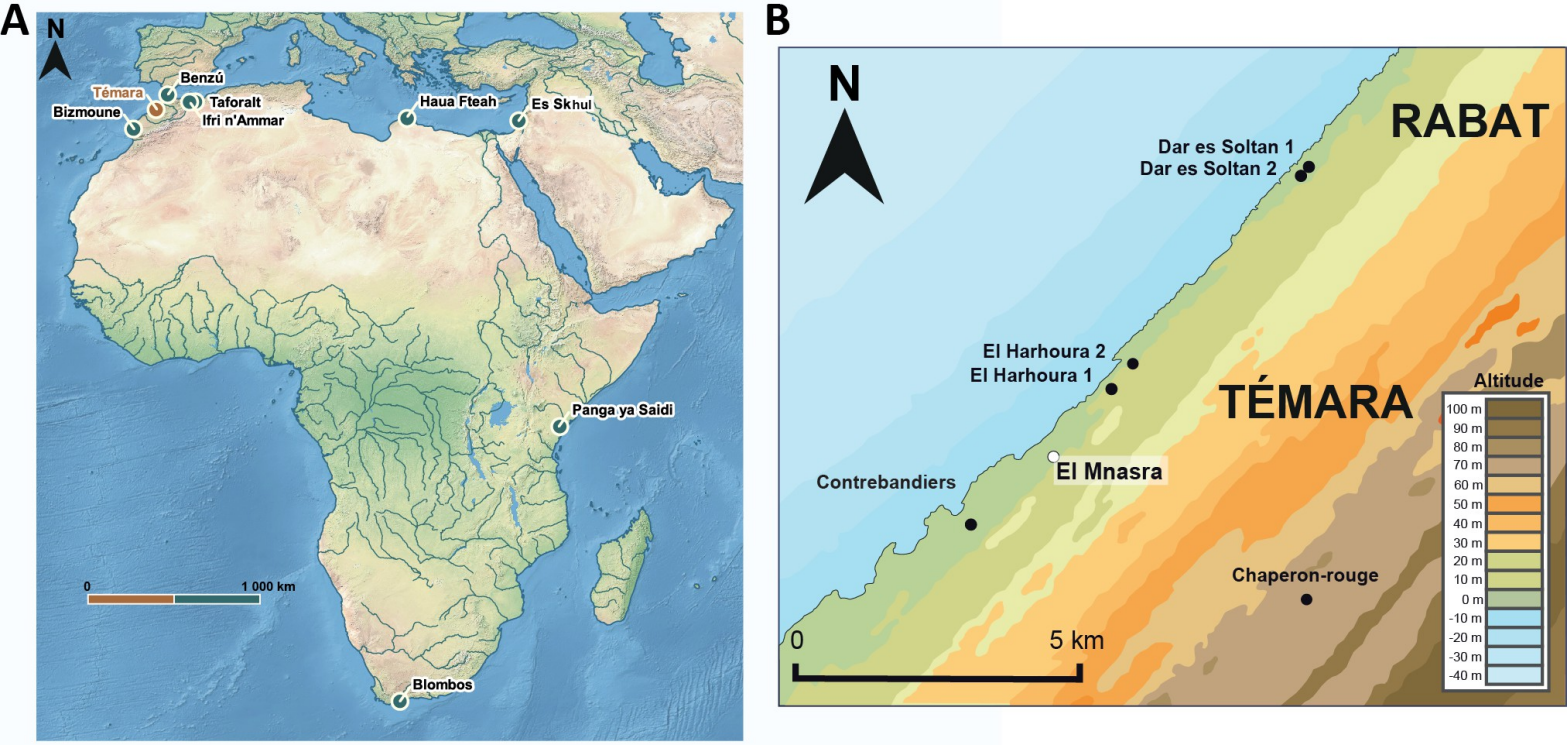
Location of the sites mentioned in the text. A: MSA sites in Africa (the map was made using QGIS software v.3.20 and using data from Natural Earth vector map data (https://www.naturalearthdata.com/downloads/)); B: MSA sites of Témara region, adapted from Ben Arous et al. (2020).

Until recently, the debate surrounding the role of exploitation of the coastal environment in human brain evolution and demography has mainly focused on MSA South African sites [8, 9]. However, many North African coastal MSA sites present evidence of marine resources exploitation (Fig 1): in Libya at Haua Fteah [14], in Morocco at Benzù rock shelter [15] and the Rabat-Témara region (Contrebandiers [16, 17]; El Mnasra [18]; El Harhoura 1 [19]; El Harhoura 2 [18, 20]). Evidence of marine exploitation is associated with Aterian Moroccan sites. Considered as an MSA techno-complex specific to North Africa, the Aterian (~ 150 ka to 40 ka, [21–27]) is defined by the presence of tanged and foliated tools in the lithic assemblages (Fig 1) [4, 28–30].

The Rabat-Témara region (Fig 1) hosts rare examples of North African stratified coastal sequences, with well-preserved MSA occupations yielding evidence of behavioural complexity and marine shells used for food consumption or ornamental purposes [5]. Outstanding research carried out at El Harhoura 2 [20] and El Mnasra cave shows a great diversity of marine shells. Two specific marine resources, Patellidae and Mytillidae, were preferably consumed and were directly collected on rocks [20]. At El Mnasra cave, the preserved MSA occupations have delivered artefacts attesting the use of hematite pigments, production of bone artefacts and Nassariidae perforated shells [4]. This site is crucial to study livelihood strategies adopted by the populations of the North African Middle Stone Age coastal areas in relation to the production of advanced symbolic and cognitive hallmarks. For these reasons, the chronology of the MSA sequence at El Mnasra is of special significance.

El Mnasra cave has already benefited from radiometric dating (S1 Table), which placed the MSA occupations embedded in the sediment from ~112 ka (MIS 5d) to ~70 ka (MIS 4). Discrepancies have been observed repeatedly between existing combined US-ESR dates [31] and OSL dates for the same US [24], but no argument has been put forward to explain it.

Recent fieldworks have also revealed the stratigraphic complexity of the site, and several biological disturbances have been observed locally, many of them related to Honey badgers (*Mellivora capensis*) or Neolithic human burials [5]. Following these new observations of the deposits sequence and the bioturbated areas, a new dating campaign was engaged to support the chronology of El Mnasra Middle Stone Age deposits.

In this paper, we present eight new dates for North African MSA occupations at El Mnasra cave: (i) two optically stimulated luminescence (OSL) dating of sediment samples to date the end of the MSA; and(ii) six U-series combined to the Electron Spin Resonance (combined US-ESR) dating of animal teeth from the stratigraphical unit with the highest MSA human occupations and evidence of behavioural innovations (e.g. Nassariidae perforated shells). The new results are integrated in the Bayesian model to provide a chronostratigraphic model, that includes new stratigraphic information and previously obtained ages.

### El Mnasra cave: Stratigraphy, lithic assemblage and existing chronology

**Fig 2:**
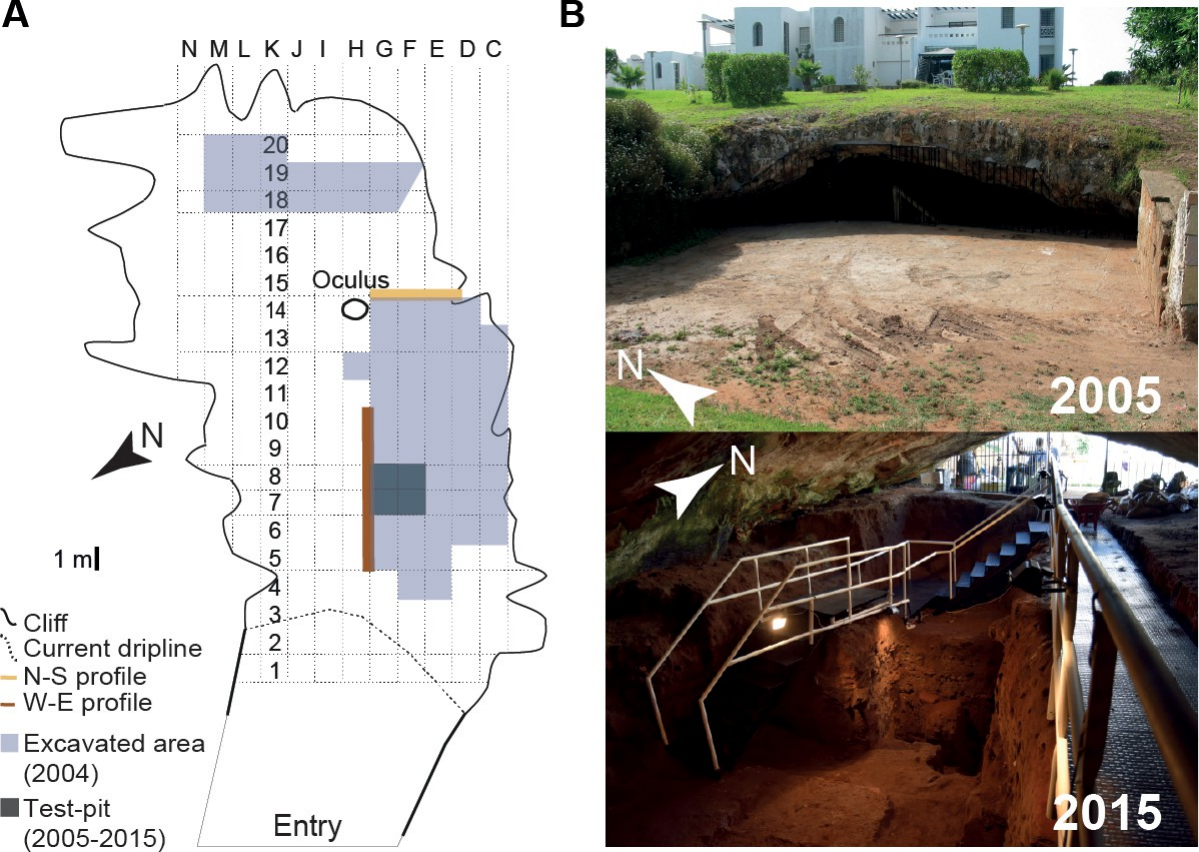
El Mnasra cave. A: Excavation map in 2015 [34]; B: view of the cave, outside (2005) and inside (2015) the cave.

El Mnasra cave (33°55’ 40.9’’N, 6° 57’ 13.3’’ W) is located in the Rabat-Témara region (delimited by Wadi Bouregreg in the northeast and Wadi Yquem in the southwest), to the south of the city of Rabat (Fig 2). The cave is shaped on a calcarenite cliff on the Atlantic coast and opens onto the Ouljian cliff formed during the high sea level of the last interglacial, ca. 130–125 ka [24, 25, 32, 33]. The substratum is formed by local Plio-Pleistocene indurated dunes, which deposition ended around MIS 5e/5c [32, 33].

Today, El Mnasra cave is located 500 m from the shoreline. The top of the cave is 14 m above the current sea level [5]. The cave is 14 m wide and 22 m long. In the cave’s roof, there is an oculus with a size of 1.00 x 0.90 m, located above the West-East profile near the back of the cave (Fig 2). The total estimated surface area of the deposits is about 230 m².

The cave was discovered in 1956 by J. Roche [5]. The first excavations were initiated by the INSAP (*Institut National des Sciences de l’Archéologie et du Patrimoine de Rabat*) between 1990 and 2000 under the direction of M.A. El Hajraoui and A. Debénath [35]. From 2005 to 2015, El Mnasra was excavated by the cooperative field project "Mission Archéologique El Harhoura-Témara" of the MNHN (*Muséum national d’Histoire naturelle*) and the INSAP, co-directed by M.A. El Hajraoui and R. Nespoulet.

### Stratigraphy, geomorphological and chronostratigraphic context

The first stratigraphical description of 4.40 m was carried from the test-pit by M.A. El Hajraoui [35] and adapted by A. Debénath in 2006 [5]. Based on lithological and archaeological criteria, thirteen archaeological levels were identified as follows, from bottom to top: levels 13 to 11 are archaeologically sterile, levels 10 to 3 are attributed to the MSA, level 2 is attributed to the Neolithic, and level 1 is disturbed and reworked. Levels 10 and 9 yielded very little archaeological material. The presence of the Later Stone Age (LSA), initially attributed to level 3, was unconfirmed by recent excavations [5].

A re-assessment appeared necessary with the extension of the excavation area, which revealed new sedimentary facies with various geometries. The relationships between stratigraphic units (US) were more complex than previously assumed with the simple reading of the section of the test pit. Supplemented by the cross-sections, the new reading places all the deposits in a single stratigraphic framework by considering the main section of ~10 meters in large and ~5 meters in depth (S1 Fig). During the excavation campaign in 2010, the sedimentary sequence re-examination presented for the first time in this paper was undertaken and recorded by A. Lenoble in the West-East profile during the excavation campaign in 2010. A preliminary version of this study was outlined as an excavation report [36], but had hitherto never been completed and published.

The new lithostratigraphy identifies six ‘*Members*’ and thirteen stratigraphic units (US). The distinction between ‘*Members*’ reflects significant changes in the geometry of the sediment, as evidenced by discordances and slope changes, while the distinction between units is based on significant changes in source or sedimentary facies. Based on the observation and precise description of sedimentary facies, we propose a new sequence reading. In this paper, stratigraphic units (or US) defined from the new lithostratigraphy reading are preferentially used, whereas "Level" refers to old stratigraphy defined by A. Debénath and M.A. El Harjraoui and will not be used (Table 1). The lithological succession of interest here (S1 Fig) is summarised in Table 1. To clearly associate previous dated samples with newly defined US, previous chronometric datings [24, 27, 31] are indicated in Table 1. With this new stratigraphic description carried out in the field, the position of some already dated samples has been revised by A. Lenoble (e.g. there are no dates associated with the US 8c and US 9a). For example, the sample EM10-5 dated by Jacobs et al. [24] was associated with level 4 (US 7), whereas the new stratigraphical reading allowed to replace it accurately in level 3 (US 6). We thus use it, we will take it as a reference for this work and subsequent studies. A complete description of the main geological/sedimentation events from the new lithostratigraphic lecture is provided in S1 Text in S1 File.

**Table 1:** Lithological description of El Mnasra.

Members	US (Level)	Description
1	US 1 (Level 1)	(0.10 to 0.80 m)—Excavated deblais accumulated in the central and rear part of the cave, coarsely stratified with lenses of sediment from the various underlying US; sharp lower boundary (erosive). A sheet of aluminium foil observed at the base of the US indicates its recent age, possibly the digging debris of Abbé Roche’s excavation.
	US 2 (Level 2)	(0.25 m)—Shell midden (*kjoenkkenmoedding* facies) preserved in the entrance part of the cave, composed of dark grey to black sediment rich in land snail and Mytilidae shells.
2	US 3 (Level 3)	(ca 0.75 m)—Dark reddish-brown loamy sand, poorly sorted, massive, forming a bowl morphology deposit cutting into the underlying unit; irregular and sharp lower limit extended downward by strong burrows of the soil megafauna (e.g., badgers) up to the US 8. This unit is interpreted as highly bioturbated sediments reworking mainly US 4 and 5 and, to a lesser extent, the underlying US 6 to 8.
3	US 4 (Level 3)	(0.2 to 1.00 m)–Reddish-brown clayey fine sand, massive, well-sorted, preserved in the rear part of the cave with a maximal thick below the oculus—[**EM-1702**^**a**^ sampled at the base of the US 4]
	US 5 (Level 3)	(ca. 0.45 m)–Reddish-brown to light reddish-brown clayey fine sand with intercalated few centimetres-thick beds of black organic clayey sand with irregulars’ boundaries punctuated by soil macrofauna synsedimentary activities; preserved lamination is less bioturbated zone indicating of a runoff deposit—[**EM10-2** sampled in an organic bed located at the middle of the US 5; **EM10-1** sampled in organic bed and located at the top of the US 5]
4	US 6 (Level 3)	(ca. 0.20 m)—Gentle domed form and apex positioned in the central part of the cave; Brown to dark grey fine sand, massive, bioturbated, including lenses of marine shells (patella) in the entrance area and lenses of anthropogenic sediments (ashes, charcoals) intersected by bioturbation channels in the rear part of the cave—[**EM-1701**^**a**^**, EM10-5** at 0.05–0.10 m above the base of the US 6]
	US 7 (Level 4)	(ca. 0.25 m)–Bedded sandy loam cemented on top with alternating light yellowish-brown and carbonated brown beds (interpreted as dominated by ashes and/or hearth cleaning); the beds appearing as lenses over several meters horizontally; domed formed inclined westward; sharp lower limit—[**EM10-6** at 0.10 m above the base of the US 7; **EM-274**^**a**^; **EM08-12**; **EM10-3** at the top of the US 7]
5	US 8	(0.75 m)–Several tens of centimetre thick beds of massive or faintly bedded brownish sand and loam with intercalated ashy or charcoal-rich lenses, dipping 6° toward the south (towards the entrance). Four main beds are individualised:
	US 8a (Level 5)	• Bed 8a (ca. 0.10 m)–massive greyish brown sandy loam including soft intraclasts at the top (trampling?), rich in snails toward the top, and faint bedding at the base; substantial archaeological fraction (charcoals and grey ash lenses or charcoal lenses topped with white ash)—[**EM08-10** at the US base; **OSL-X2416; EM-209**^**a**^**; EM08-11**];
	US 8b (Level 6)	• Bed 8b (ca. 0.40 m)—Faintly bedded red-brown clayey sand; the bedding is related to chromatic variation (red-brown to dark brown bands). Locally, pure sand beds; otherwise, scattered archaeological fraction (charcoals, shells, ash aggregates) gradually decreasing topward— [**EM08-8** at 0.10m above the base of the US; **EM0604; EM-293**^**a**^**; EM0603; EM-223**^**a**^, **EM08-9**];
	US 8c (Level 7)	• Bed 8c (ca. 0.15 m)—Massive greyish brown to dark brown clayey sand; intercalated charcoal or ash lenses; scattered archaeological elements;
	US 8d (Level 7)	• Bed 8d (ca. 0.15 m)—Massive brown clayey loams including charcoals and scattered soft intraclasts; a few interspersed ash lenses and reddish-coloured ash and sediment aggregates; incipient bedding in relation with intercalated reddish bands—[**OSL-X2415, EM08-7, EM0601, EM-816**^**a**^**, EM-877**^**a**^].
	US 9	Several ten centimetres-thick beds of light brown to brown loamy to clayey sand and loam, dipping 3° toward the southwest, truncated by gullying in places, some archaeological elements in the mid part of the unit. Six main beds are recognised, with only the first four (US 9a to 9d) on the reference section:
	US 9a (Level 8)	• Bed 9a (ca. 0.10 m)—Bed of light beige loams (ashes) cemented at the base, rising gently into the cave, finely laminated and topped with beds of beige-grey and black laminated sandy clay;
	US 9b (Level 9)	• Bed 9b (ca. 0.15 m)—Several centimetres thick beds of slightly sandy brown, reddish-brown or greyish clay with scattered charcoals and rare lenses of ashes—[**EM08-6**];
	US 9c (Level 10–11)	• Bed 9c (ca. 0.15 m)—Powdery light yellowish-brown carbonated loam, lenticular bedding with plane lenses of charcoal topped with white ashes (interpreted as hearths) and lensoidal mixed ashes rich in aggregates of burned sediment; numerous archaeological elements—[**EM08-3, EM08-4, EM08-5**];
	US 9d (Level 12)	• Bed 9d (ca. 0.35 m)–Three to ten centimetres thick lenticular beds of brown to red-brown clay; these beds can crosscut at a low angle; carbonated elements (shell fragments); some charcoals and aggregates of burnt sediment in the median bed exhibiting an aggregate structure—[**OSL- X2414** at the middle of the US 9d; **EM08-2**];
	US 9e (Level 12)	• Bed 9e (up to 0.30 m)—Gully infilling intercalated between US 9d and US 9f, with a net erosive base, containing either a succession of black and beige clayey silt beds or an accumulation of angular aggregates of clay taking up these materials from the underlying clayey sediment;
	US 9f (Level 12)	• Bed 9f (ca 0.15 m) is similar to 9d.
	US 10	(0.45 m)—Horizontal beds are ten centimetres thick of brown to yellow-brown fine clayey sand with puddle filling facies with intercalation of a settling deposit at the base. Three main beds:
	US 10a (Level 12)	• Bed 10a (ca. 0.20 m)—Yellow-brown to brown decametric thick beds of fine sandy clay or sandy clay with a massive structure. Locally underlined by a dark centimetric bed (manganese enrichment?);
	US 10b (Level 13)	• Bed 10b (ca. 0.10 m)—Well bedded (laminated) unit formed by a succession of black and beige one to several centimetres thick beds, finely laminated or exhibiting a mixed structure (bioturbation) according to the places of observation (bioturbation). Presence of finely laminated rills filled with sandy clay;
	US 10c (Level 13)	• Bed 10c (ca. 0.10 m)—Medium to fine sands very slightly clayey, faintly bedded, with darker and discontinuous centimetre thick lamination at the base. Massive structure at the top. A sharp base is locally underlined by a 3 cm thick bed of light clay and fine black laminations passing laterally to a mixed structure (bioturbation?).
6	US 11 (Level 13)	(0.10 to 0.50 m) Moderately sorted, massive, carbonated, medium sand including rare marine shells of centimetric size (beach deposit) and some elements of microfauna. Yellow-brown colour progressively darkening in the upper 15 cm and correlative acquisition of a poorly expressed lamination by 1) sorted infracentimetric intercalation and 2) intercalated brown clay-loam laminations—[**OSL-X2413; EM08-1** at 0.10 m above the substratum]

US are correlated to the archaeological Level, and we indicate the correspondence between US (new lithostratigraphy) and Level (previously published stratigraphy) as followed: US (Level). OSL and combined US-ESR samples are indicated in brackets; ^*a*^: new samples analysed in this study. Note that with the revision of the position of the samples in the new stratigraphy [21, 23, 39], the US 8c and US 9a are no dated.

### Cultural assemblages

**Fig 3:**
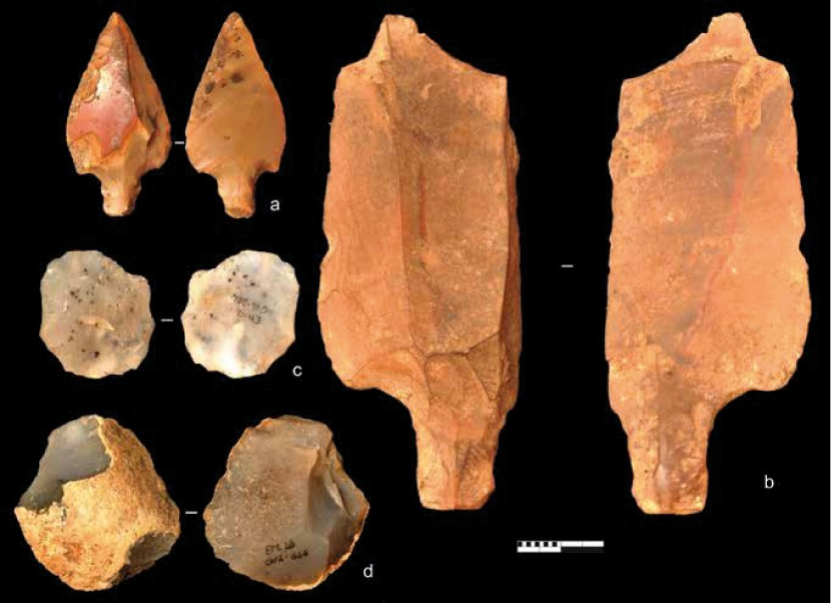
Aterian MSA lithic industries from US 9a. a, b: tanged tools with a: retouched tanged point; b: retouched Levallois blade; c: Levallois micro-blade; d: Levallois micro-nucleus; scale: 1 cm (© R. Nespoulet).

**Fig 4:**
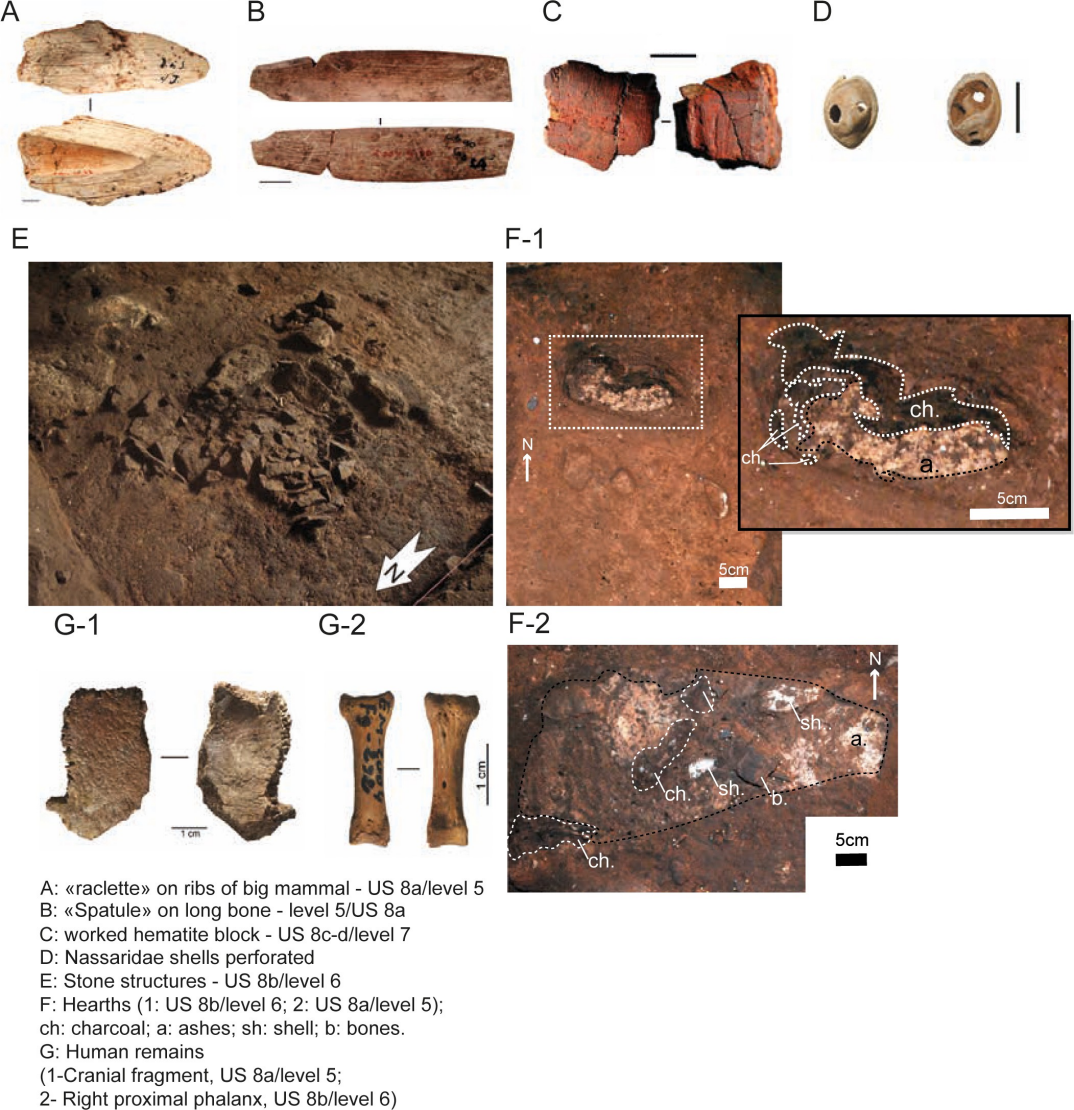
Material evidence of cultural/behaviour innovations and human remains found at El Mnasra cave.

The lithic typological data available come only from US 10a to US 7 [5]. To our knowledge, there are no publications on the lithic assemblages of the US 6, 5, 4 and 3. Until 2015, US 10a to US 9a were only known from the main test-pit at the front of the current excavation area (Fig 2A). A few archaeological materials, such as fragment of a nucleus or pigment fragments in these deeper US could indicate an early human presence in this cave (Table 1). MSA industries of El Mnasra are characteristic of the Aterian culture (Figs 3 and 4), with the presence of tanged tools, Levallois/micro-Levallois debitage (flakes and cores), and side-scrapers [5]. El Mnasra also yielded evidence of bone tools [4] and pigment use in an Aterian MSA context in archaeological levels 5 to 7 [5, 37]. In addition, the cave has delivered a large number of marine shells associated with combustion areas and, to our knowledge, the largest number of Nassariidae (N>236) associated with a MSA context in Africa [5, 38]. Hearths are known in archaeological levels 5 and 6, some of them delimited by stone structures [5, 35], others characterised by the presence of intense combustion areas clearly delimited by limestone pavements in archaeological levels 6 and 8 [5, 35]. Human remains were found in levels 5 and 6 (Fig 4).

### Previous chronology

El Mnasra cave has already benefited from fairly radiometric dating with a total of 25 dates obtained before this study (S1 Table) and in the test-pit sector. First, four single aliquots Optically Stimulated Luminescence (SA-SAR OSL) ages on quartz grains were obtained by Schwenninger et al. [27]. In 2012, a new large-scale chronological study led by Jacobs et al. [24] obtained eighteen single quartz grain OSL ages (SG-SAR OSL). These previous OSL ages placed the MSA occupations embedded in the sediment from ~112 ka (MIS 5d) to ~70 ka (MIS 4). The application of the combined US-ESR method in 2012 [31] has shown that all three ages are younger than OSL dates for the same US. These discrepancies have been observed repeatedly, but no argument or discussion has been put forward to explain them. Furthermore, the previous three US-ESR ages were calculated with one gamma dose-rate measurement and one sediment. A set of charcoal and burned seeds were collected in hearth and ashes areas from US 9a and US 8 [39] and dated by the AMS-radiocarbon method and are all beyond the limits of radiocarbon dating (> 50 ka), confirming their antiquity. An attempt to date occupations at the top of the sequence by the AMS-radiocarbon method [40] and OSL [24] failed due to bioturbated context. As a result, the upper part of the cave deposits could never be dated. Following the excavations from 2012 until 2015 in the main excavation area in parallel with a new reading of the deposits, new dental material from large mammal remains was unearthed, making new chronological applications possible to date the MSA occupations directly.

## Material and methods

### Combined US-ESR dating

The combined US-ESR method can be applied to fossil remains to directly date hominin or animal remains [41]. Tooth enamel contains hydroxyapatite and thus can be used as a natural dosimeter, and ESR can determine its accumulated dose. In parallel, U-series data are obtained on the different tissues constituting the tooth (all of which incorporate uranium (U) during burial) and combined with the ESR data. This coupling allows determination of the timing of uranium uptake thanks to the calculation of the uptake parameter, the ’*p-value*’ determined for each tissue [42]. The *p-value* ranges from -1 (corresponding to an early uptake, EU) to positive values, corresponding to a recent uptake or RU [43]. The combined US-ESR method is used for samples that did not experience U-leaching. Such leaching can result in overestimated U-series ages. Leaching is indicated by ^230^Th/^234^U ratios in dental tissue > 1 or by U-series age > EU-ESR age [42].

#### Sampling, samples preparation and equipment

For combined US-ESR dating, six Bovidae teeth stored at INSAP (EM-274, EM-209, EM-223, EM-293, EM-877, EM-816; S2 Fig and S2 Table), well preserved in appearance, were selected. The sampling considered the current spatial configuration of the excavation and the material available. These teeth originate from US 7, 8a, 8b, 8c and 8d. ESR samples were prepared following the standard ESR dating protocol [22, 44] and analysed at the Geochronology laboratory (MNHN, Paris). The preparation protocol is detailed in S2 Text in S1 File.

Three supplementary teeth (EM0601, EM0603 and EM0604), previously analysed by Janati-Idrissi et al. [31], are from US 8b and 8d. Their ages were recalculated thanks to the new dose-rates measurements provided in this work, leading to new ages.

#### ESR dose evaluation

The evaluation of the equivalent dose is based on the multiple aliquot additive dose (MAAD) method. The protocol of the ESR dose determination is detailed in the S2 Text in S1 File. All ESR dose response curves (DRC) with detailed fitting results are provided in S3 Fig.

#### Dosimetry

**Fig 5:**
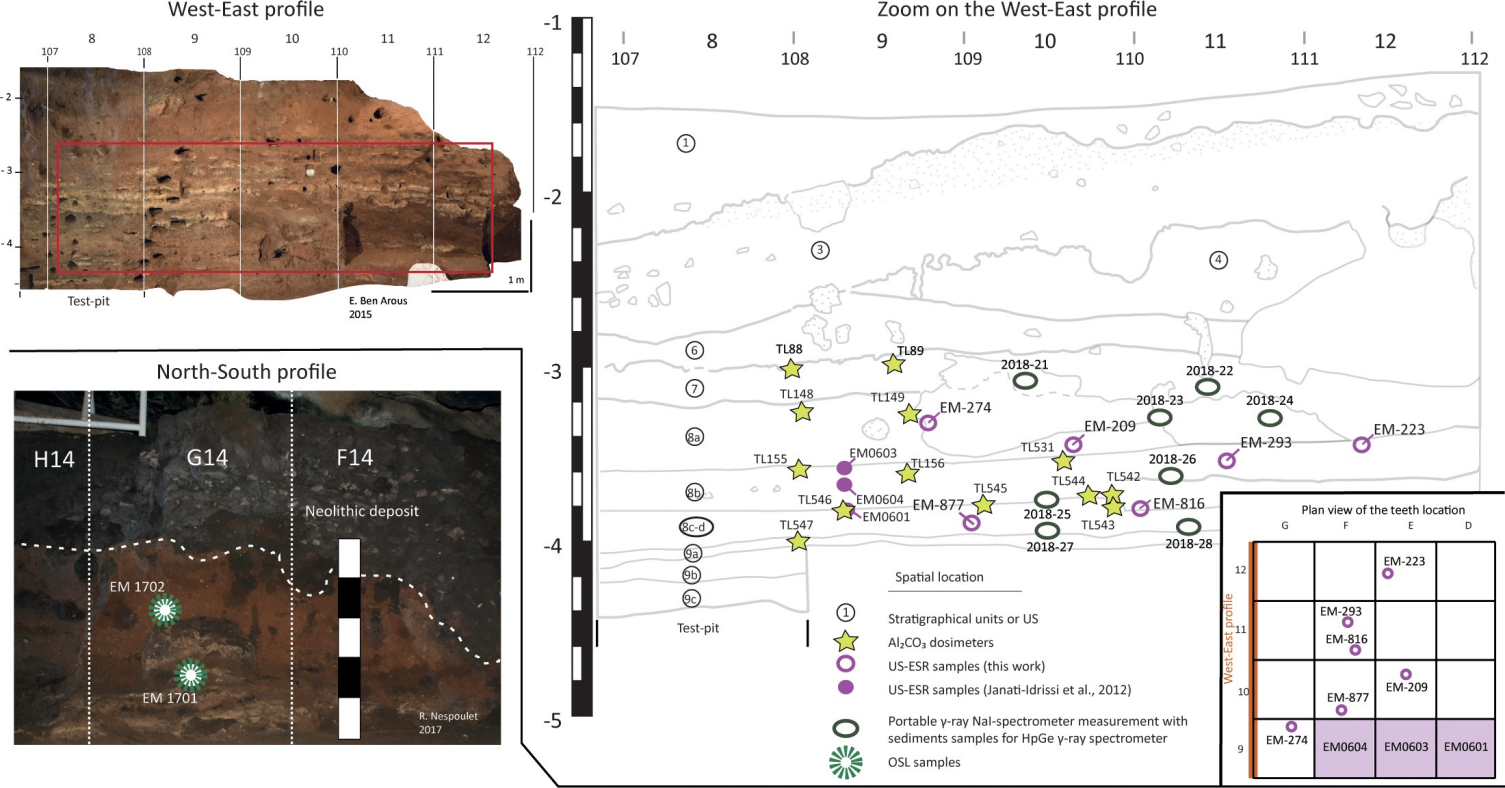
Location of OSL and combined US-ESR (altitudinal projection) samples analysed in this work. This spatial representation is an altitudinal projection that can lead to a slight apparent offset of the sample locations and the US slops on the profile. The location of the teeth is also showing in the plan view. For the teeth EM0601, EM0603 and EM0604, the squares are indicated in purple.

One of the difficulties in combined US-ESR dating is the determination of γ and β-dose-rates received by the teeth. They calculate from the radioelement concentrations of the sediments and it is impossible to measure the doses at the precise location of teeth used for dating. To overcome this problem, eight sediment samples from the West-East section were collected from US 7 to 8d, from which the teeth originated, and taken for subsequent laboratory analyses. Sediment samples were conditioned in plastic bags to preserve their moisture. The spatial location of all samples is represented in detail in Fig 5.

Dosimetry in ESR dating of tooth enamel consists of the internal α- and β-dose-rates within the dental tissues, and external components from β- and γ-radiation of radioelements from the teeth encasing/surrounding sediments, plus a cosmic dose. The determination of the dosimetry for the Combined US-ESR dating is detailed in the S2 Text in S1 File.

#### Age calculation

Combined US-ESR ages were calculated with the following parameters: α-efficiency of 0.13 ± 0.02 for enamel [21], a water content of 5 ± 3 wt% in dentine and cement, while 0% were assumed for enamel. The water content of the sediment was measured in the laboratory by drying at 40°C for ten days, and estimated from the weight difference sediment before and after drying. Eight sediments presented low water contents, between 3.3 wt% to 11.4 wt%. The water contents used to calculate the β dose-rates for each US are average data, presented with their standard deviation and ranging from 5.7 ± 0.5 to 7.8 ± 2.0% (S5 Table). α and β-attenuation factors used for enamel follow Brennan et al. [22]. The cosmic dose rates values considered are the same as those presented by Jacobs et al. [23], calculated from the equations of Prescott and Hutton [24]. They include correction considering the site altitude, geomagnetic latitude, density and thickness of cave rock (2.5 g/cm^3^) and sediment (1.8 g/cm^3^) overburden, and the cos²_ϕ_ zenith angle dependence of cosmic rays. Values are ranging from 150 ± 20 μGy/a (US 8c/d) to 160 ± 20 (US 8c to US 7).

Combined US-ESR ages (1σ) were calculated using the DATA program [45] with error calculations derived from Monte Carlo simulations. The sample geometry cement/enamel/dentine was used for the beta dose rate attenuations for EM-223 and sediment/enamel/dentine configuration was used for the other samples.

### Optically stimulated luminescence dating

Optically Stimulated Luminescence (OSL) dating method was employed to date quartz minerals and the sedimentary context (strictly the event(s) of bleaching and subsequent burial). During the burial, energy is trapped in quartz grains through time. OSL uses an intense light source to release and measure this energy initially absorbed by the material, the equivalent dose (D_e_). Measurements performed on multi-grains aliquots provide a distribution of equivalent doses (D_e_). However, they do not allow identifying individual problematic grains (signal saturated grains, incompletely bleached), which is crucial in the context of a cave, especially when bioturbation is identified. To identify such grains, single-grain (SG) measurements were therefore carried out.

#### Sampling, samples preparation and equipment

In the West-East section, the sediments are extensively reworked by bioturbations, and the state of excavations between 2008 and 2012 did not make it possible to clearly identify US 6 to US 3. US 7 was already dated by single-grain OSL in this section (Fig 5). Nevertheless, two sediment samples (S2 Table) were collected on the North-South section in the MSA US 6 (EM-1701) and the sterile US 4 (EM-1702), the distance between the two samples being about ca. 0.40 m. Our new single grain OSL dates allow us to better constraint the chronology of the last occupations at the cave and the taphonomy of the rest of the sequence. Both sediments were collected using opaque plastic tubes (25 cm in length and 5 cm in diameter), sealed with adhesive tape and an aluminium cover, and packed in black light-opaque bags to preserve the light-sensitive OSL signal during transport. The chemical preparation and the measurement implementation are indicated in S3 Text in S1 File.

#### Equivalent dose determination

Preliminary tests were carried out in the laboratory on single aliquots and detailed in S3 Text in S1 File. In order to discard the grains which were unable to properly record the D_e_, the Central Age Model (CAM) was applied with the progressive removing of grains with the lower D_0_ values to obtain mean D_e_ values no longer influenced by D_0_ values [46–48]. Subsequently, D_e_ are recalculated for subsamples of full distribution of D_e_ by discarding the smallest D_0_. This process allows to obtain a plateau: the beginning of the plateau indicates that the mean D_0_ no longer influences D_e_ values. This "threshold test" was carried out in this work on the D_e_ obtained in single-grain mode (S5 Fig). To account for the possibility of mixing phases in El Mnasra sediment, the Finite Mixture Model (FMM) was tested [49, 50] by running 2–4 discrete dose components using the R package “RLumShiny” [51, 74].

#### Dose rate determination and age calculation

Gamma-dose-rates (field and laboratory) were measured following the same protocol presented for US-ESR dating. In laboratory, ca. 10 g of sediment samples, previously dried at 40°C during five days, homogenised, crushed and sealed in plastic tubes were measured by High-purity germanium (HpGe) γ-ray spectroscopy with a high-resolution detector Canberra-Eurisys well-type detector at the IRAMAT-CRP2A laboratory. In this study, the quartz grains were not etched and the alpha contribution was considered. For the 100–140 μm fraction, we used the α attenuation and β absorption factors following Brennan et al. [52] and Guérin et al. [53], respectively.

OSL ages presented with 1σ have been calculated by the ANATOL software (version 2.0.6), considering dose-rate conversion factors from Guérin et al. [53]. As for the US-ESR age calculations, the cosmic dose-rate for both samples was chosen following the value of 170 ± 20 μGy/a as provided by Jacobs et al. [24]. Water content was measured and determined at 5.8 ± 0.6 wt% and 2.8 ± 0.3 wt% and considering a matrix water saturation of 30 ± 15% and 15 ± 7% for the sample EM-1701 and EM-1702, respectively.

### Bayesian age modelling

A hierarchical chronostratigraphic model of the El Mnasra sequence derived from Bayesian event modelling was built using Chronomodel V2.0.18 [54]. The main purpose of the Bayesian model is to develop a global dating of all the US by including prior information on the samples dated. The model implemented by Chronomodel combines several varying individual dates (*t*_*i*_), individual uncertainties and information based on stratigraphy to estimate the age of the target event (θ), here the new stratigraphic unit with preserved MSA occupations. The discrepancy between *ti* and θ is modelled by an individual variance (σ^2^_i_) that allows the model to be robust to outliers and does not require additional analysis to remove them from the data set. Three recalculated US-ESR ages (see section Results and Discussion) are considered to yield terminus post quem ages and should be interpreted as a minimal age of samples in chronological modelling: as this is not allowed in the chronomodel software, these three ages have not been included in the Bayesian modelling.

We used Bayesian modelling to propose a global model that conciliates the chronologies based on our OSL and US-ESR ages published by Schwenninger et al. [27], Jacobs et al. [24], and the OSL and US-ESR dates obtained in this work. In this Bayesian approach, each dated event is estimated from its posterior distribution calculated from its likelihood function and prior information. We can then characterise a temporal period defined by a group of dated events (often called phase).

First, we calculated a time range interval for each US modelled as a Phase, which characterises the period during which a Phase happened. Time range intervals represent the specified posterior probability (95% and 68%) of containing all samples ages affected to the US [55]. Secondly, the posterior density of the Start and End ages for each US is represented by their density curves, corresponding to the associated error. No supplementary boundaries have been used to constrain the dates for the lowest US 11 and the uppermost US 4.

30 numerical dating results obtained using OSL and US-ESR methods have been included as likelihoods in El Mnasra Bayesian model. The luminescence and ESR numerical results were input into the model with their associated 1σ uncertainty ranges. Dating data are nested in US (Phases) with succession constraints between them: all the samples dated at a same US are older than those in the US above it, and vice-versa.

We build the Bayesian model considering two independent data sets for illustrative purposes: 24 OSL ages and 6 US-ESR ages separately. Finally, we run Bayesian modelling by combining OSL and US-ESR ages.

Markov Chain Monte Carlo (MCMC) algorithms known as the Metropolis-within-Gibbs strategy is implemented in Chronomodel to approximate the posterior distributions of ages. Three Markov chains are simulated in parallel. For each chain, 1000 iterations are used during the Burn-in period, 20 batches of 500 iterations are used in the Adapt period and 100 000 iterations are drawn in the Acquire period.

## Results and discussion

A detailed presentation of the US-ESR measurements results is described in the S4 Text in S1 File and in S4–S6 Tables.

### Combined US-ESR ages

In total in our study, six US-ESR ages were obtained (Table 2) and three ESR ages were updated from data provided in Janati-Idrissi et al. (2012). Final ages were calculated using average radioelements contents (S4 Table), and we provided ages calculated with average external γ-dose-rate (S6 Table). *P-values* (S7 Table) describe the kinetic of uranium incorporation in each dental tissue [41] and indicate if the latter has significantly changed over time. *P-values* for dental tissues are higher than −1 (between -0.55 and 0.52). ^230^Th/^234^U ratio is lower than 1 (between 0.224 and 0.399, S4 Table), suggesting no uranium leaching in the dental tissues.

**Table 2:** US-ESR ages were calculated with γ-external reconstructs with gamma *in situ* dosimetry and with α-Al_2_O_3_:C dosimetry.

Sample	US	D_e_ (Gy)	±	D_(α + β)_ (μGy/a)	±	D_β1_ (μGy/a)	±	D_β2_ (μGy/a)	±	D_γ_ (μGy/a)	±	D_β (ext.)_ (μGy/a)	±	D_cos_ (μGy/a)	±	Ḋ (μGy/a)	±	US-ESR ages (ka)	±
EM-274	7	47.4	1.8	3	1	30	3			356	14	129	16	160	20	678	29	69.9	4.0
EM-209	8a	40.3	1.2	2	1	25	4			316	36	127	28	160	20	630	50	64.0	5.4
EM-223	8b	40.2	0.9	2	1	7	1	17	3	325	35			160	20	511	55	78.7	8.6
EM0603*	8b	38.2	0.7	24	8	31	5	32	5	325	35			160	20	572	41	66.8	4.9
EM-293	8b	45.2	1.3	1	1	12	1			325	35	89	9	160	20	587	56	77.0	7.7
EM0604*	8b	44.2	2.8	11	5	13	3	13	3	325	35			160	20	522	51	84.7	9.9
EM0601*	8d	37.2	0.7	31	10	18	4	20	6	340	62			150	20	559	66	66.5	8.0
EM-877	8d	47.1	0.9	5	1	13	2			340	62	60	9	150	20	568	67	82.9	9.9
EM-816	8d	49.7	1.7	5	2	12	2			340	62	64	8	150	20	571	82	87.0	12.8

Equivalent dose (De), Dose-rate and combined US-ESR age estimation presented at 1 σ confidence level. Key: e = enamel; d = dentine; c = cement. The dose-rate components presented are: (D(α+β)—internal dose-rate of the enamel; Dβ1—beta contribution from the dentine; Dβ2—beta contribution from cement; Dβ (ext.)–beta contribution from sediment; Dγ - gamma external dose-rate from sediment; Dcos–cosmic dose-rate. *: US-ESR ages recalculated from Janati-Idrissi et al. [24].

Combined US-ESR ages follow an apparent global stratigraphic order. EM-274 from US 7 is dated at 70 ± 4 and 64 ± 6 ka for EM-209 from US 8a. Considering the associated 1σ errors, they are all indistinguishable. Samples from US 8b provided older ages, ranging between 77–85 ka. The dose-rate value for sample EM-293 (587 ± 561 μGy/a) is higher than that calculated for samples from the same US. This difference mainly comes from the dental tissues, particularly the internal enamel dose-rate and the β-dose-rate from cement of EM-223, EM0604 and EM0603. The ages of the MSA occupations in the US 8c/8d are between 83 ± 9 and 87 ± 10 ka. The probability intervals of 1σ ages cover a chronological range from MIS 5c to MIS 4.

Three ESR ages from teeth samples (EM0601, EM0603, EM0604) published by Janati-Idrissi et al. [31] were updated and compared to our data. These previous data were calculated with one inserted TL dosimeter in US 8b and using one sediment to calculate the β-dose-rate from sediment. We recalculated the ages these authors published, considering (i) the *in-situ* measurements performed within US 8b in this present and previous work, (ii) the mean radioelement contents of the sediments taken in this present work. The recalculated ages (Table 2) are in the same time interval as the other teeth. However, in details, the 2/3 US-ESR samples (namely EM0603 and EM0601) are younger than the surrounding US-ESR samples for the same US. The internal dose-rate is one of the significant differences between the three teeth samples and those newly analysed in this work. There is a factor of 10 difference between the U-contents in the enamel of new samples (ca. 0.01 ppm) and those of Janati-Idrissi et al. (ca. 0.1 ppm) obtained by α-spectrometry analyses, while the contents in the dentine or cement are similar in both studies. This factor of 10 lead to an internal dose-rate overestimated for EM0601, EM0603 and EM0604. This explains why EM0603 and EM0601 are younger than others teeth for a given layer. EM0604 gave comparable results with other teeth for the US 8b. Because of the uncertainties for 2/3 samples previously dated, we consider that these are minimum ages.

### Luminescence measurement

The OSL measurements results are described in the S5 Text in S1 File.

Two OSL single-grain ages and associated dating details are shown Table 3. Dose-rate for each sample is provided in S7 Table. For EM-1702 a total dose-rate of about 1441.7 ± 12.7 μGy/a was determined, which is 18% higher than EM-1701.

**Table 3:** OSL results.

Sample	EM1701	EM1702
US	6	4
De (Gy)	57.72 ± 6.46	80.05 ± 4.58
OD (%)	72 ± 8	30 ± 5
K (%)	0.63 ± 0.016	0.67 ± 0.014
U (ppm)	1.19 ± 0.26	1.447 ± 0.027
Th (ppm)	3.9 ± 0.09	3.787 ± 0.075
Moisture content (%)	5.8 ± 0.6	2.8 ± 0.3
α (μGy/a)	23.1 ± 4	26.2 ± 3.7
β (μGy/a)	669.6 ± 33.5	754.3 ± 10.8
γ (μGy/a)	324 ± 32	491 ± 5.6
Cosmic (μGy/a)	170 ± 20	170 ± 20
Total (μGy/a)	1186.8 ± 46.5	1441.7 ± 12.7
Age (ka)	48.63 ± 6.87	55.52 ± 5.28

Results obtained using the Central Age Model (CAM) with the "threshold test" show a plateau for D_0_ values around 80 Gy and 68 Gy (S7 Fig): the D_e_ selected for the sample EM-1701, and EM-1702 are 57.72 ± 6.46 Gy and 80.05 ± 4.58 Gy, respectively.

Critically, for both samples, FMM showed no discrete D_e_ component representing the majority of the grains in either of the two samples and untangle the D_e_ distribution. FMM is, therefore, not the most appropriate model. Any further interpretation of the current data would be speculation, and a more in-depth investigation will help understand this type of distribution. Then it is more reasonable to retain the CAM age at 1σ instead to avoid any bias. Considering the CAM, sample EM-1701 is dated at 48.63 ± 6.87 ka, and sample EM-1702 is dated at 55.52 ± 5.28 ka. Considering the 1σ errors, the two ages are relatively close.

### OSL and combined US-ESR chronologies comparison

**Fig 6:**
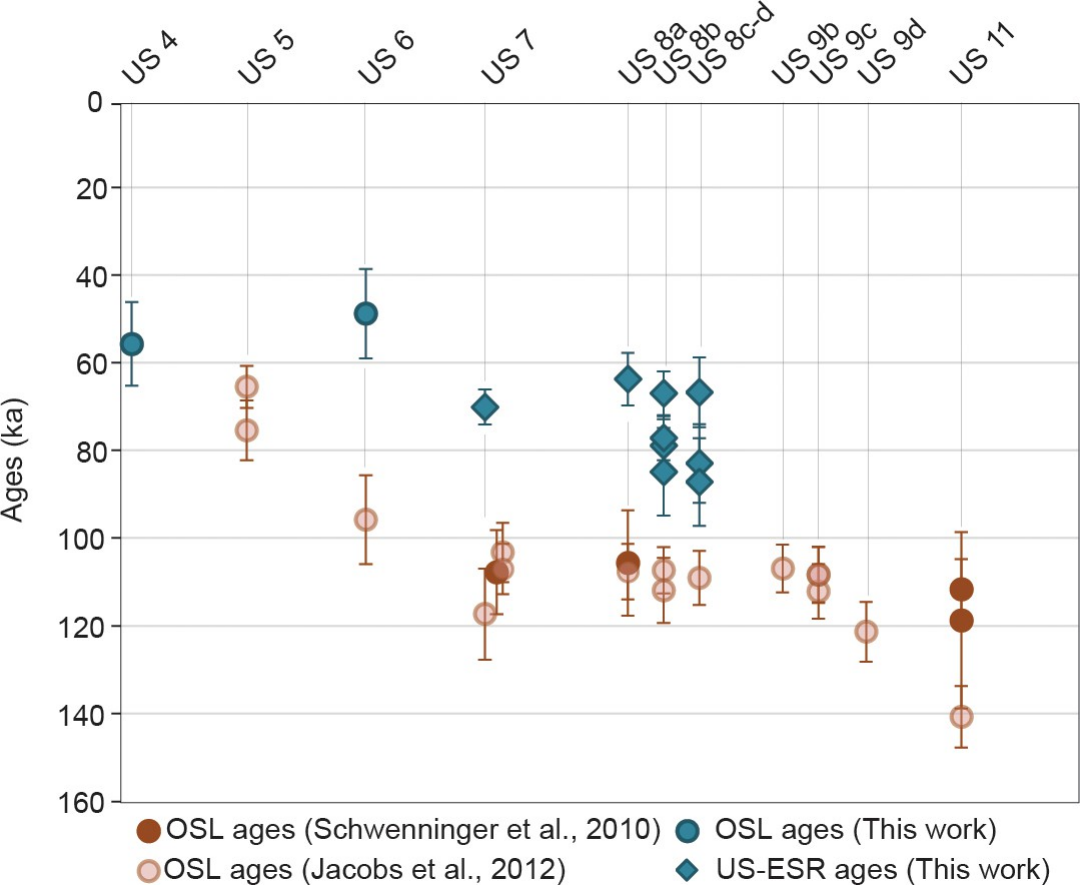
OSL and US-ESR ages available for El Mnasra cave. Errors are displayed at 1σ.

All ages available for El Mnasra cave are represented in Fig 6. First, combined US-ESR ages are at least 38–49% younger than OSL single-grain [24] and multi-grains ages [27] for the same US (7, 8a, 8b, 8c/d). Secondly, the CAM age for the US 6 is almost 50% younger than the age of the sample EM10-5 (S1 Table) and placed in the same time range of the sample EM10-2 (US 5) and sample EM1701 (US 4). In addition, CAM ages provided in this work are coherent and close to the US-ESR ages for the uppermost US 7.

Several sources of uncertainty could explain the origin of this discrepancy between chronologies based respectively on combined US-ESR and OSL ages. A first hypothesis would be that some teeth could be reworked from their original context. Although this hypothesis is plausible, all dated teeth were carefully selected and well contextualised during the excavations in the area where archaeological material was found *in situ*.

We then explored the evaluation of the annual dose as the first factor of uncertainty. Uranium incorporation represents at most 5% in the dental tissue analysed in this work and up to ~15% for previously analysed teeth. Our US-ESR data did not show uranium leaching into dental tissue. Consequently, the uranium incorporation has minimal impact on US-ESR ages. Cosmic-ray dose-rate represents ~23–31% of the annual dose: it is reasonable to assume that this parameter can influence the US-ESR and OSL ages calculation. However, this parameter has been standardised according to the values given by Jacobs et al. [23] and then, do not explain the discrepancy between OSL and US-ESR ages.

Thirdly, we explored the evaluation of the environmental dose coming from sediments. For example, we suspected an impact of the seasonal change in humidity in the cave. Many authors of this paper (E. Ben Arous, D. Richter, R. Nespoulet, C. Falguères, A. Lenoble, E. Stoetzel) have noted that lawn above the cave is regularly watered and possibly contributed to the circulation of water from the oculus (Fig 1) to the deposits above. This leads to an increase of the moisture in the West-East section in squares 7 to 11. We could consider in the first instance that the water content measured in the sediment is unlikely to be an accurate estimate of the long-term water content. There is a substantial variation of water in the sediments measured by Jacobs et al. [23], leading to 35 ± 9 wt values for the upper US. We performed age sensitivity tests by varying the long-term water content from 5 to 35% to possibly quantify the impact on our US-ESR ages (S10 Fig). There is a non-significative increase of our ages, as the maximum variation is < 7% (~5 ka).

The gamma dose-rates account for 50–64% of the external dose-rate value. The gamma dose-rate determined in this work and its comparison with the available data provided in Table 3 show consistent values at 1σ. If the US-ESR ages are recalculated using the extreme gamma dose-rates values (i.e., the smallest value and the largest value for each US, Table 3), this results in age variations with a non-significative impact of ~5% at the total for US 7 to significant age variations for US 8 (~15% for US 8a, ~18% for US 8b, and up to 35% for US 8c/d).

The discrepancy between previous OSL ages and combined US-ESR ages could also be explained by underestimating the D_e_ values obtained for the fossil teeth due to unstable radicals in the hydroxyapatite as already observed [56, 57] on enamel fragments. However, ESR dose reconstruction was carried out in the present study on homogeneous enamel powder, which results in the randomly spatial distribution of the enamel fragment. This hypothesis remains unlikely: this underestimation was demonstrated on tooth fragments from the Middle Pleistocene, which are extremely rich in uranium and therefore have a different internal contribution from the teeth analyzed in this work. Furthermore, Dirks et al. [58] showed that this phenomenon might not affect all teeth analyzed by the combined US-ESR method.

The hypothesis of incomplete bleaching of the quartz grains resulting from remobilization of a sedimentary stock present in the cave or extremely close to the cave entrance (S1 Text in S1 File) cannot be entirely excluded and could explain the D_e_ overestimation for the OSL ages. The sediment deposition processes in the cave are extremely complex and result from many factors, as highlighted by A. Lenoble (S1 Text in S1 File). For example, the sediment deposits of the US 10 to 8 (US 8 is the most important MSA occupations in terms of material density) represent the product of remobilization by runoff from a coastal sedimentary stock mainly represented by aeolianites in a secondary position. Therefore, it is still challenging to understand the influence of such depositional processes in the OSL dating of quartz grains. Further micro-morphological studies in the future will undoubtedly provide interesting elements for discussion. Informatively, the previous OSL ages calculated using the FMM [24] indicate that grains with D_e_ with a major component chosen represents between 64.3 ± 13.3 to 97.5 ± 1.2%, while the minor components represent about 1 ± 0.6 to 35.5 ± 14.3% of the dated grains [24]. The recalculation of the OSL ages considering the different components of D_e_ determined to result in ages for US 7 to US 8c-d closer to the US-ESR ages (S11 Fig).

### Bayesian model and time range intervals

**Fig 7:**
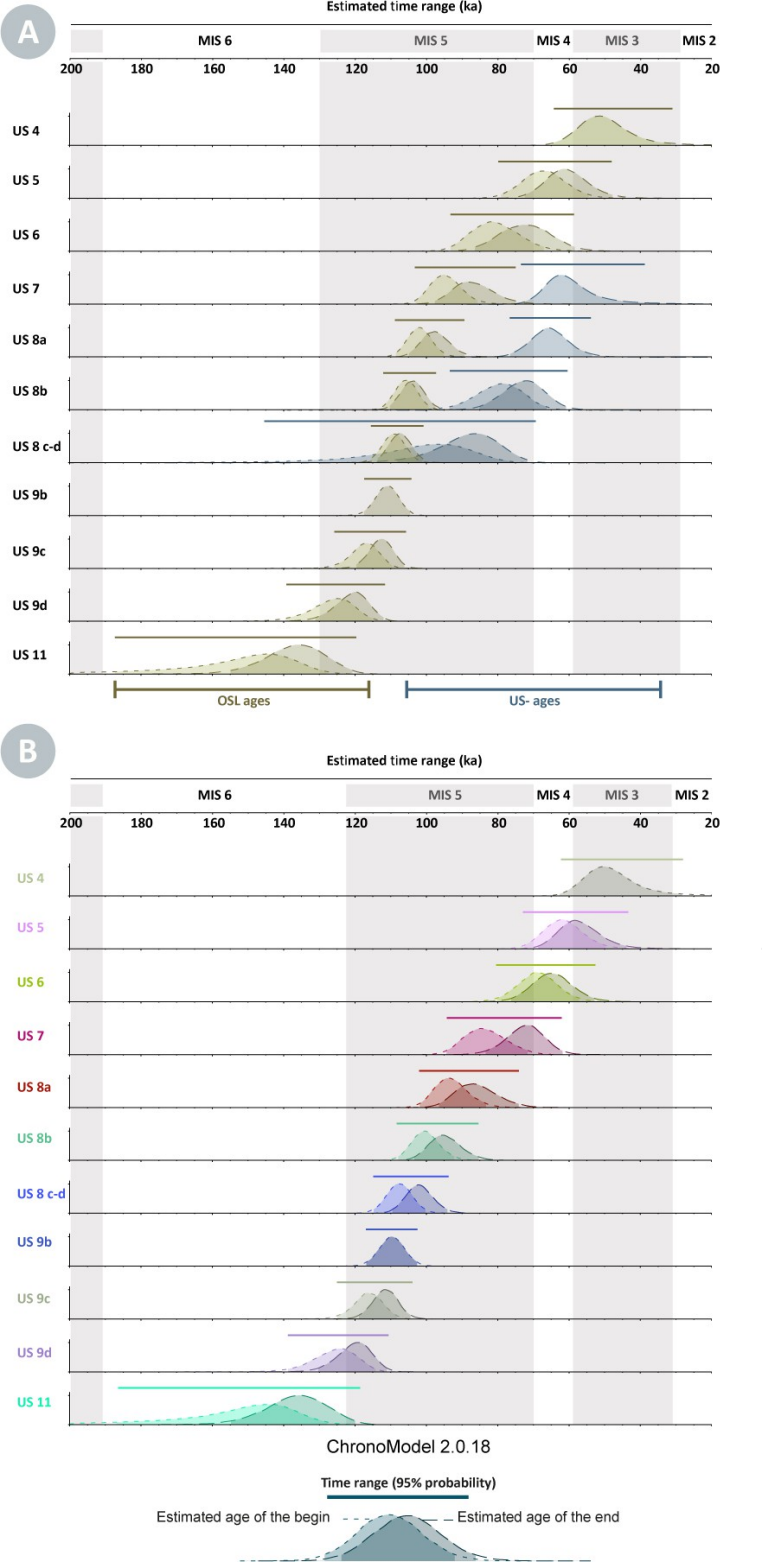
Bayesian model results of the 30 ages (OSL, combined US-ESR age) used to constrain the age of the US obtained with Chronomodel v.2.0.18). A: the Bayesian modelling independently considers OSL and US-ESR data; B: Bayesian modelling combines all data. Start and end ages have been estimated for each US and the curves of posterior densities are represented. All ages are shown in thousand years (ka) before present.

The results of the Bayesian modelling are summarised in Table 4 and Fig 7. Time range intervals are rounded to the closest century. Each modelled Phase (US) is associated with time range intervals and reported in 68% and 95% probability. A posterior density curve represents the Start and End ages for each US. No supplementary boundaries have been used to constraint the time range for the lowest US 11 and the uppermost US 4. All OSL and US-ESR samples within each Phase are nested with the robust cross-correlations of sedimentological-lithological boundaries between the Phase-US.

**Table 4:** El Mnasra 95%- and 68%-time range intervals calculated with OSL data, US-ESR data and combining all the data.

**OSL ages**	**ESTIMATED TIME RANGE (ka)**			
	**68% probability**		**95% probability**	
	from	to	from	to
US 4	58	44	64	31
US 5	73	55	80	48
US 6	88	66	93	59
US 7	100	83	103	75
US 8a	106	94	109	90
US 8b	109	101	112	98
US 8c-d	112	104	115	101
US 9a				
US 9b	114	108	117	104
US 9c	121	109	126	106
US 9d	131	116	139	112
US 10a				
US 10b				
US 10c				
US 11	160	125	187	120
**US-ESR ages**	**ESTIMATED TIME RANGE (ka)**			
	**68% probability**		**95% probability**	
	from	to	from	to
US 7	67	55	74	39
US 8a	71	61	77	54
US 8b	85	66	94	61
US 8c-d	111	76	146	70
**All ages**	**ESTIMATED TIME RANGE (ka)**			
	**68% probability**		**95% probability**	
	from	to	from	to
US 4	56	43	62	28
US 5	68	52	73	44
US 6	74	59	80	53
US 7	89	67	94	62
US 8a	99	82	102	74
US 8b	105	91	108	86
US 8c-d	111	98	115	94
US 9a				
US 9b	113	106	117	103
US 9c	120	108	125	104
US 9d	130	114	139	111
US 10a				
US 10b				
US 10c				
US 11	160	124	186	119

This modelling aims to provide a Bayesian modelled chronology considering both OSL and US-ESR dates. Therefore, we present the final Bayesian modelling, which incorporates the two data sets (Fig 7). US 9e to US 10c are not covered by sampling. Consequently, it makes no statistical sense to model time intervals without dates. US 9e-US 9f are channels that intersect with the US 10a but not the US 9d: they are necessarily younger than the time range interval of the US 10a and posterior to the time range interval of US 9d. The time range above and below allows it to be framed and considered as minimum and maximum intervals.

The resulting representation by independently considering OSL and US-ESR ages shows an expected discrepancy with the un-modelled OSL and US-ESR chronologies. In detail, modelled time range using US-ESR ages for the US 7 to 8 c-d are generally younger than those obtained solely with OSL ages (Fig 6). Secondly, the main effect of considering all data has been to provide longer intervals than if we only considered US-ESR and OSL data for US 7 to 8c-d. Considering the 95% probability, the time range interval with combining all ages is around 14%, 44%, 56% and 44% (respectively for US 7 to 8c-d) longer than interval modelled by solely OSL ages. Conversely, the time range intervals modelled solely with US-ESR ages are more precise of about 8%, 32% and 78%, respectively for US 7, 8b and 8c-d. Finally, stratigraphic constraints on ages have little effect on US 9b and US 11: the time intervals that consider all data are 10%, 6% and 2% longer than those considering only OSL ages. Reasonably, we take into account the model which combining various dates because (i) it includes all the data from the different studies, (ii) its remains more robust to uncertainties and inconsistencies, and (iii) the good robustness properties of the event date model are counter balanced by a lower precision in the dates for US 7 to 8c-d but compensated by a more reliabile chronology overall.

### Chronology and MSA occupations model at El Mnasra cave

**Fig 8:**
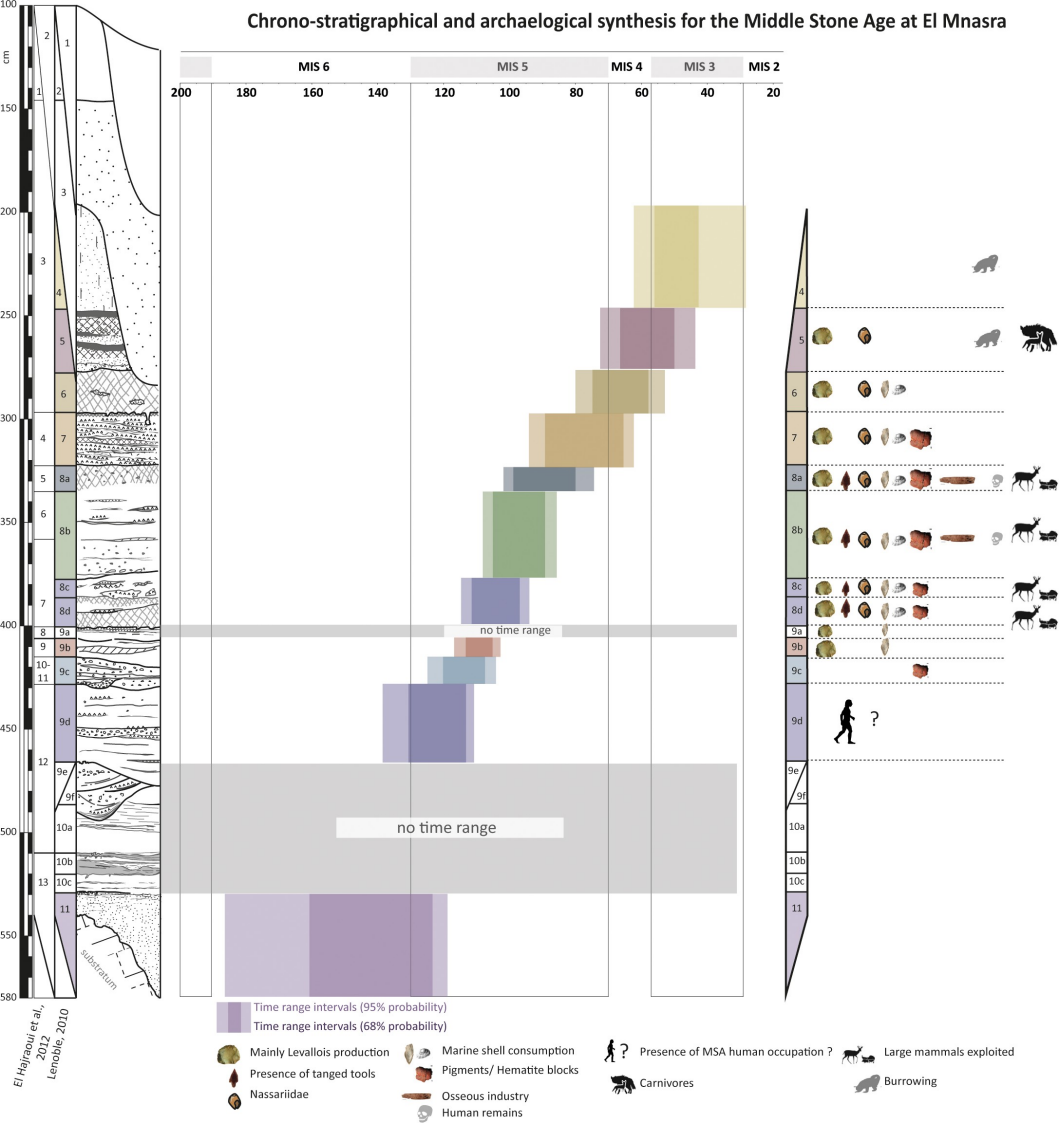
Synthesis of the Middle Stone Age from El Mnasra cave. Time range intervals are indicated in front of each US (Lithostratigraphical log). Archaeological materials for each US are also indicated. Adapated from Stoetzel et al. (2014b).

The time range intervals (95% of probability) were added to a synthesis considering a synthetic section of the cave deposit, archaeological and paleoenvironmental data available for El Mnasra cave. We have also considered the sparse paleoenvironmental data extracted from large and small vertebrates [5, 18, 34, 38, 59–63]. These data are synthesised in S9 Table. Fig 8 summarises all available archaeological and the time range intervals with 95% probability as presented in Table 4.

According to the lithological description and interpretation (S1 Text in S1 File), the sterile US 11 is a coastal beach deposit trapped in the cave and associated with a high sea level of the MIS 5e. Bayesian model confirms this observation by placing the edification of the US 11 between 186–119 ka.

**Fig 9:**
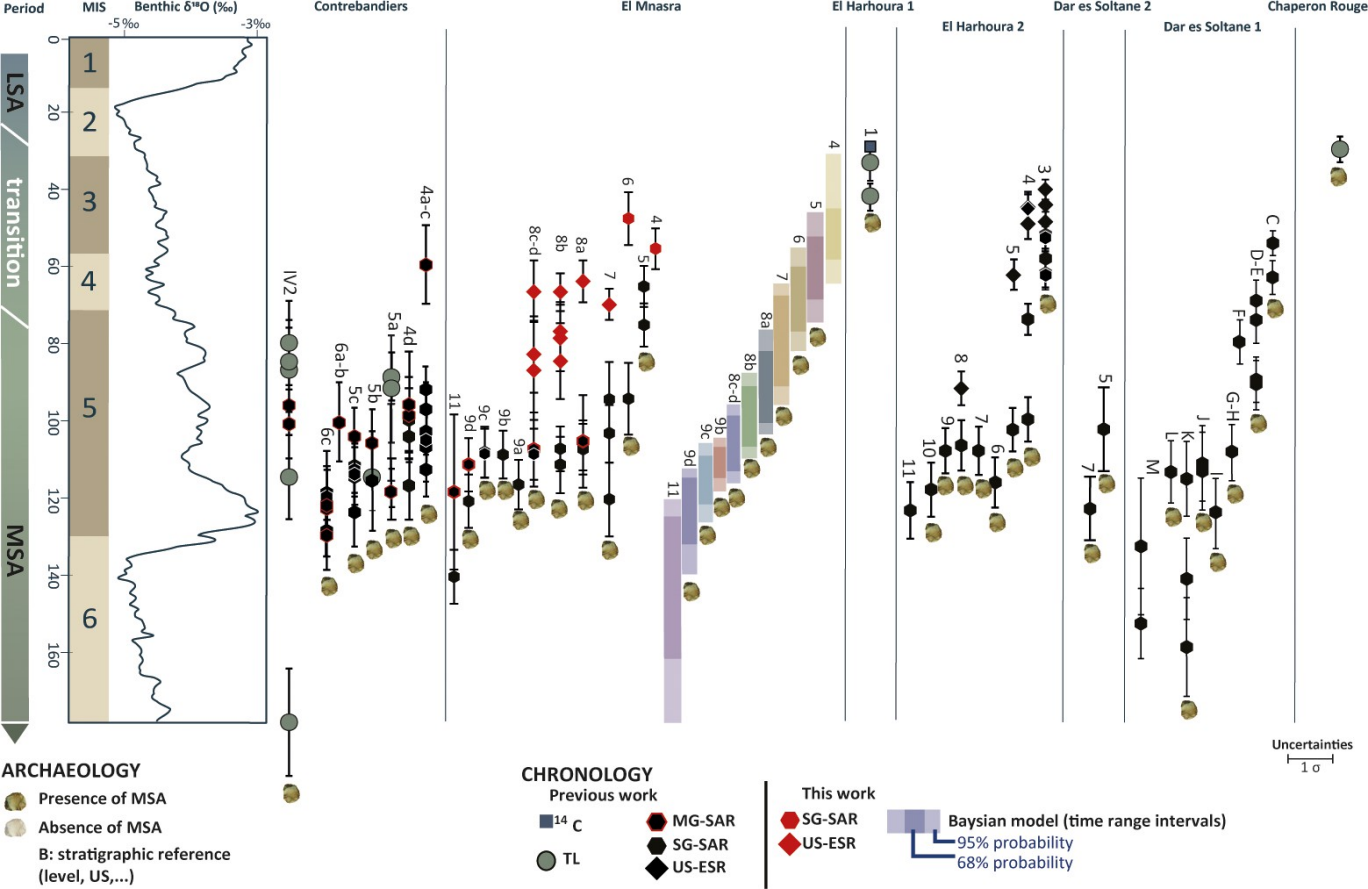
MSA chronological synthesis of Témara’s caves considering previous dates and new dates and time range intervals provided in this paper for El Mnasra cave. Dates placed in the same vertical lines belong to the same stratigraphical unit or level.

The oldest evidence of human presence is attested in the US 9d by anthropogenic sediments visible in section. We provide a period estimation between 139 and 111 ka for this US, pushing the lower limit of the MSA to MIS 5d. The US 9c presents also very clear anthropogenic facies delimited in the section, with laminated charcoals and ashes beds. Artefacts interpreted as archaeological materials, namely fragments of hematite and stone tools [5] found in US 9c are placed between 125 and 104 ka. This is relatively similar to the oldest MSA occupations preserved at the neighbouring caves (Fig 9) of Dar es Soltan 1 (112 ± 8 ka)[27, 32] and Contrebandiers caves (level 6c, weighted mean age of 122 ± 5 ka [25]).

As the lithological reading of the US 8d to US 8a shows the absence of the marine contribution in their sedimentary edification (S1 Text in S1 File), there are probably placed around the high sea level of the MIS 5d [64, 65]. Zooarchaeological studies [18] from MSA occupations of the US 8 (US 8d to US 8a) show that North African Aterian populations at El Mnasra have exploited a diversity of marine molluscs (Patellidae, Mytilidae, Trochidae and Muricidae, [18]). Two specific marine taxa, Patellidae and Mytillidae, were preferably consumed. They are intertidal molluscs living on a rocky substratum and likely accessible during low tides near the cavities [38]. A study in the nearby cave of El Harhoura 2 showed the presence of characteristic notches on Patellidae shells, suggesting that they were directly collected on the rocks by MSA human groups [20]. Evidence of marine resources collecting and consumption could indicate that the sea level was close to the current one at these periods and that the coastline was very close to the cave’s entry: this is therefore consistent with the attribution of the sedimentary edification to the MIS 5c. For these US, the faunal spectrum is slightly diversified and mainly dominated by *Gazella dorcas/atlantica*, including *Bos primigenius*, *Equus* sp., *Connochaetes taurinus* and/or *Oryx* cf. *dammah* [5]. Microvertebrates and large mammals from US 8b suggest that the climate was relatively humid and that the landscape was dominated by savanna-like environment, with sparsely wooded areas [34, 61, 62]. Time range intervals place the US 8c-d, 8b and 8a between 115–94 ka, 108–86 ka and 102–74 ka, respectively.

Patellidae and Mytilidae were found in abundance in the US 7 and US 6 [38], clearly visible in section and, during the excavations, by well-delimited shell beds [38]. One of our hypotheses attributes US 7 and/or US 6 to MIS 5a, around 80–70 ka. Time range for these two US provided by Bayesian modelling is consistent with this hypothesis, placing the US 7 and US 6 between 94–62 ka and 80–53 ka.

According to geomorphological observations, deposits of US 5 and US 4 are characterised by a clear continental component and were ascribed to MIS 4/3 or MIS 2 (S1 Text in S1 File and Table 1). The presence of organic soils in US 5 suggests preserving vegetation in an environment with aeolian sediment accumulation (semi-arid environment) from the MIS 4, whereas their absence in US 4 could indicate complete desertification of the landscape. Lithological interpretation of the US 4 agrees with the microvertebrate assemblages: the presence of jerboas (*Jaculus* sp.), today absent from this region, indicates an open and arid environment (dry and rocky steppes), but the presence of a relatively good diversity in amphibians indicates the persistence of soft waterbodies close to the site. The geomorphological interpretations and faunal data could agree with the model, time range intervals are 73–44 ka and 62–28 ka. However, it was impossible to further constrain the end of US 4 due to the dearth of reliable dates for the uppermost US 3. Therefore, we cannot exclude the possibility that the upper limit of US 4 around 30 ka could reflect a statistical artefact. Preliminary zooarchaeological studies associated to the MSA human occupations of US 5 showed that non-human predators seem to be the main occupants of the cave, alternating with short human occupations. Many carnivore marks attest this on the faunal remains and the low presence of lithic tools [60].

It is impossible to discuss US 3 and US 2 because no material suitable for dating has yet made it possible to date them.

Based on the previous chronology [24], three probable periods of MSA occupations have been identified approximatively around ~110–95 ka (MIS 5c), ~80 ka (MIS 5a) and ~70 ka (MIS 4). The first two episodes displayed intense occupations, whereas the last corresponds to a low human occupation associated with non-human predator activities [60]. Occupations initially attributed to the MIS 5c and MIS 5a are marked by coastal marine exploitation and the hypothesis of their relation with the high sea levels has been explored further [60]. Our model for the MSA timeline covers all the MIS 5 and MIS 4 and brings more nuance, showing that such direct association is not apparent.

Regionally, the discrepancy between dating methods is not specific to El Mnasra cave: differences between TL and OSL ages have already been found at Contrebandiers (Fig 9) and between OSL ages at Dar es Soltane 1 (Fig 9). However, similarities exist between El Mnasra cave and the others caves.

Contrebandiers cave yielded malacological spectra similar to El Mnasra with Patellidae and Mytilidae [17, 66] in levels 5–6 (Mousterian MSA) and levels 4, IV-2, V-1, V-2 (Aterian MSA). Based on OSL single-grain ages, these levels are dated between ~120 and 90 ka (Fig 9). At El Mnasra, time ranges calculated with a Bayesian approach show a similar conclusion: a constant presence of marine shells consumption from MIS 5e-MIS 5d (US 9b, 112–98 ka) to MIS 5a (US 6, ~76–61 ka).

Occupations recorded in US 9b to 5 have yielded marine shell use and consumption. However, Nassariidae perforated shells are only present in US 8d to US 5 [38]. The abundance of Nassariidae shells in US 8 deposits could be explained by the attractivity of the marine resources in the Témara region for Aterian populations around the MIS 5c to 5a. To our knowledge, the most ancient perforated marine gastropod shells were dated, in Africa, from the late Middle Pleistocene (>~ 145 ka) at Bizmoune cave [67]. Elsewhere in Morocco, they were dated around 88–78 ka at Ifri n’Ammar [26, 68], ~82 ka at Taforalt [69] and around 75 ka at Blombos cave [70]. Outside Africa, they were dated around 135–100 ka at Es Skhul in Israël [7]. At El Mnasra cave, we provide chronological constraints for the largest African Nassariidae shell beads assemblage associated with an MSA context between ~115–94 ka (US 8c-d) to ~73–44 ka (US 5), ending at the MIS 4/3 boundary. Red pigment residues have been associated with perforated Nassariidae shells and an ongoing study (M. Lebon, pers. comm.) shows hematite in their composition. Moreover, colourant blocks have been discovered in US 8a-d and US 7 and some of the block pigments display artificial sub-parallel scrape marks [5]. Here, we provide a period estimation for the occurrence of these hematite fragments, between ~115–94 ka (US 8c-d) and 94–62 ka (US 7). The presence of Nassariidae perforated shells in many African sites suggests an interlinking exchange system [67, 69]. The influence of climatic changes could be implicated in the disappearance of the Nassariidae of the archaeological records postdating MIS 4 [71].

We propose for the first time an extended age for MSA occupations at El Mnasra cave. The younger end of the MSA in this work contrasts with Contrebandiers cave and Dar es Soltan 2, for which MSA assemblages are not persisting after MIS 5–4 according to luminescence ages [25], whereas sediments with MSA tools dated to MIS 4–3 (60–40 ka) have been found at El Harhoura 2 and Dar es Soltan 1 [22, 24, 32] (Fig 9). The MSA human groups would have briefly occupied these caves after the MIS 4/MIS 3 [4]. The absence or rarity of human occupations in the caves after the MIS 4 could thus be related to fluctuations in sea level, and the recording of coastal occupations in caves is probably incomplete [72]. Does the exploitation of coastal environments during the MSA [18, 38] suggest that human populations followed the coastline during this low sea level ? May these o sites (open air or caves) therefore be currently below sea level? It would still be very hazardous to propose only this hypothesis, given the great variability of terrestrial species consumed in El Mnasra cave [60] (S9 Table), the high mobility strategies of the Aterian groups, and their complex socioeconomic organisation [4]. It would be more likely that there existed other passage areas further inland and that the coastal sites of the Témara region were part of a larger network of occupation sites between the inland and the coastal areas as attested by volcanic caves of M’Tsogatin discovered in 2012 and located of about 100 km away from Temara region [73]. To date, our knowledge of such sites is limited, as no research has provided key information about the chronology of MSA human occupation in coastal and inland areas and the areas between them.

## Conclusion

The present study proposes a chronostratigraphic model of the Middle Stone Age occupations at El Mnasra cave with a new stratigraphy assessment and a new contextualisation of the dating samples. This work has shown the difficulties with the ages of individual samples (e.g. the precision of the dates, the question of the average γ-dose-rate representativeness). It also showed a visible discrepancy between un-modelled OSL and US-ESR dates. Further dating investigation will be crucial in refining the chronology at the top of the sequence. However, our chronostratigraphic Bayesian model using Chronomodel is a statistically robust tool that considers various data and adds stratigraphic constraints. It proposes a compromise between the different chronologies based on the OSL and US-ESR ages and offers a great consistency with lithostratigraphic, geomorphological, and archaeological interpretations for El Mnasra cave.

With the revision of the lithostratigraphy, we confirm a human presence between 124–104 ka, earlier than what the previous OSL and US-ESR data showed. Future studies and excavations will make it possible to better characterise these human occupations, and to refine the population dynamics model in Témara for the MIS 5. This is especially relevant for the Aterian in Northwest Africa, which is now placed at the first part of the MIS 5 and demonstrates that the emergence of the Aterian (and with it, behavioural and social changes) was not a response to rapid climatic/environmental changes at the end of the MIS 5 or the beginning of the MIS 4.

Our time range intervals allowed us to extend the age of the MSA occupations considerably to the MIS 4/3 boundary (~62–30 ka), marked by the disappearance of the Nassariidae perforated shells. Outstandingly, our model pushed back the age of the largest record of Nassariidae perforated shells and placed the age of their use by the Aterian groups at El Mnasra from the MIS 5d-5b (~115 and 94 ka)

This new chronological model for El Mnasra is consistent with the data from the other caves and thus makes it possible to show that regionally, the MSA is present from the MIS 5e-5d to the MIS 4/3.

The singular and interdisciplinary works at El Mnasra cave allowed a better cultural, climatic, and now temporal characterisation of the cave sequence. The implications of the new chronostratigraphic model are far-reaching for the human occupation of the region and the whole of Northwest Africa, as they raise questions regarding the participation of North African populations in dispersals inside Africa and beyond. El Mnasra and the other coastal sites of the Témara region show that the reasons for the disappearance of MSA innovation markers are still challenging to understand. It requires refining the climatic and behavioural resolution models to better understand population dynamics and behavioural variability in coastal and surrounding areas.

## Supporting information

S1 Table Previous dates available for El Mnasra cave [39]. *: The age of these sediments was recalculated according to the equivalent doses selected and indicated by Jacobs et al. (2012) in Table 2 of the article. AMS-14C ages are the uncalibrated ages. Stratigraphy from Debénath, 2006 cited in El Hajraoui et al. [5].
(XLSX)

S2 Table OSL and combined US-ESR samples dated in this work. *: US-ESR samples recalculated from Janati-Idrissi et al. [30].
(XLSX)

S3 Table Comparison of the global mean equivalent doses to the individual equivalent doses derived from each independent series of measurements. The dispersion estimated in % corresponds to the difference between the high and low De per serie.
(XLSX)

S4 Table U-series samples and data presented with 2σ error. (XLSX)

S5 Table Radioelement activities (dpm/g) from HpGe γ-ray spectrometry of dry sediments collected at El Mnasra and convert in ppm. The teeth corresponding to these measurements have been indicated. Sed-223: sediment directly associated to tooth EM-223 when it was taken from the collection. The averages calculated contents for these US are in blue and associated with standard deviation. A sedimentary description of the sediment and area sampled is given.
(XLSX)

S6 Table El Mnasra dosimetry. (XLSX)

S7 Table P-values calculated for each US-ESR samples. e = enamel; d = dentine; c = cementum.
(XLSX)

S8 Table Finite Mixture Model (FMM) details. (XLSX)

S9 Table Faunal and Paleoenvironnemental synthesis for El Mnasra cave. (XLSX)

S1 Fig Lithostratigraphy of the El Mnasra cave established by A. Lenoble. The indicated number refers to the eleven US.
(TIF)

S2 Fig El Mnasra dental samples dated by combined US-ESR method. Photos: E. Ben Arous.
(TIF)

S3 Fig ESR dose response curves (DRCs) computed by Origin Pro 8 software using Single Saturating Exponential (SSE) function. Fitting details are indicated on each DRC.
(TIF)

S4 Fig Grain size on bulk sediment decarbonated sediment and decarbonated sediment without organic material. (TIF)

S5 Fig De evolution with progressive elimination of the grains characterised by a low D0. (TIF)

S6 Fig De evolution as a function of Dmax for all samples fitted with a SSE function. 1 σ errors are displayed.
(TIF)

S7 Fig Proportion of the different US-ESR dose-rate components. Key: Internal = dose-rate α + β contribution from the enamel, β1 = beta contribution from the dentine, β2 = beta contribution from cement or β (ext.) = beta contribution from sediment.
(TIF)

S8 Fig Cut-heat test combined with the DRT for the sample EM-1701 for a standard PH temperature at 260°C. Each point corresponds to an average of 3 aliquots.
(TIF)

S9 Fig Radial plot and kernel density plot of equivalent dose distributions of single grains for samples EM-1701 and EM-1702, generated using RLumShiny package [71, 74]. (TIF)

S10 Fig Sensitivity tests by varying the long-term sediment water content from 5 to 35%. (TIF)

S11 Fig Previous single grain OSL ages calculated with the FMM components presented in Jacobs et al. [24]. (TIF)

S12 Fig Diagram presenting the relation between dates–“events” assigned to US–“Phases”. The arrow indicates stratigraphic constraints. The position of the OSL and US-ESR ages are given in the Table 1. A: OSL ages; B: combined US-ESR ages; C: global with all ages.
(TIF)

S1 File (DOCX)
