# Supplementary figures and images for: An improved chronology for the Middle Stone Age at El Mnasra cave, Morocco
Source: PLoS One. 2022 Feb 11;17(2):e0261282. doi: 10.1371/journal.pone.0261282 (PMC8836329; doi:10.1371/journal.pone.0261282)

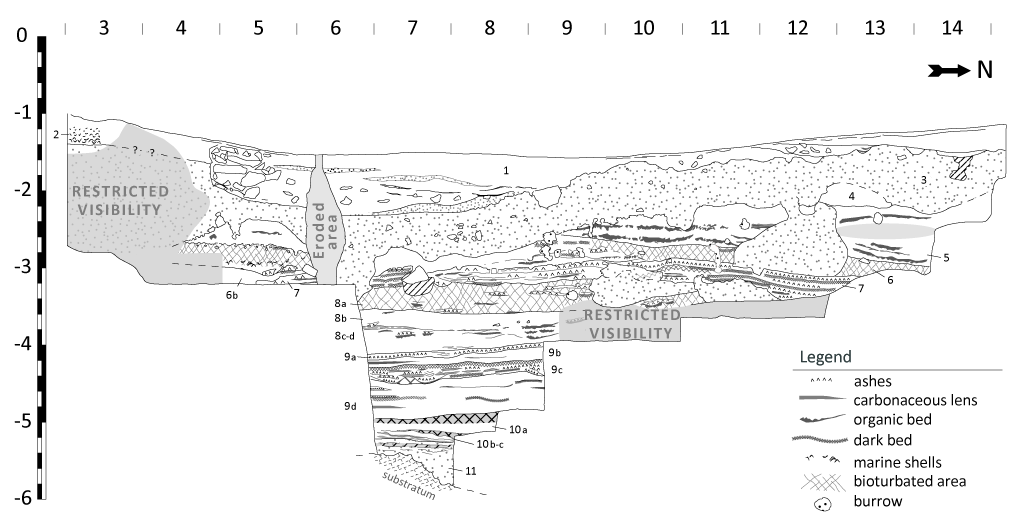

Supplement: S1 Fig — The indicated number refers to the eleven US. (TIF) [file pone.0261282.s010.tif]

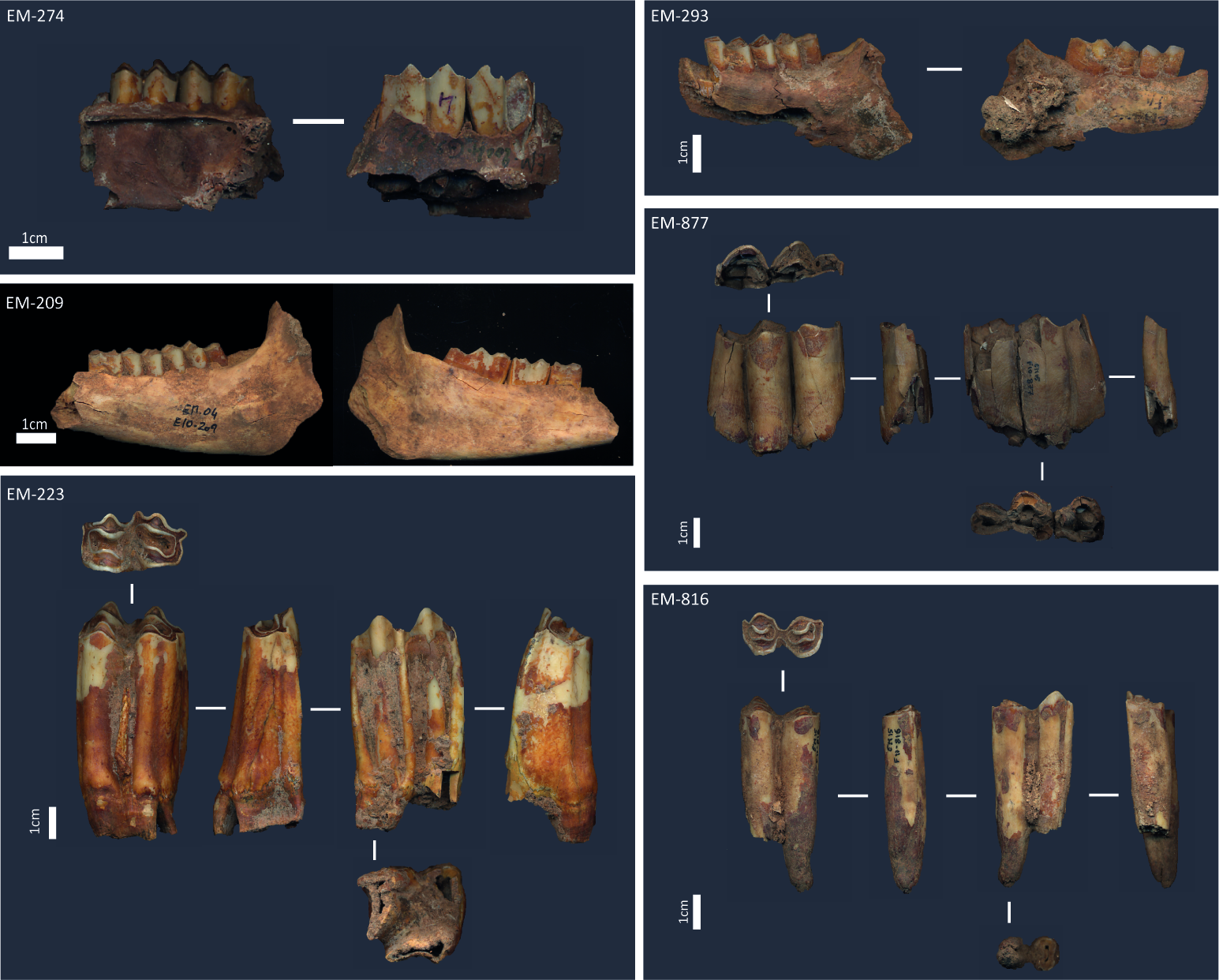

Supplement: S2 Fig — Photos: E. Ben Arous. (TIF) [file pone.0261282.s011.tif]

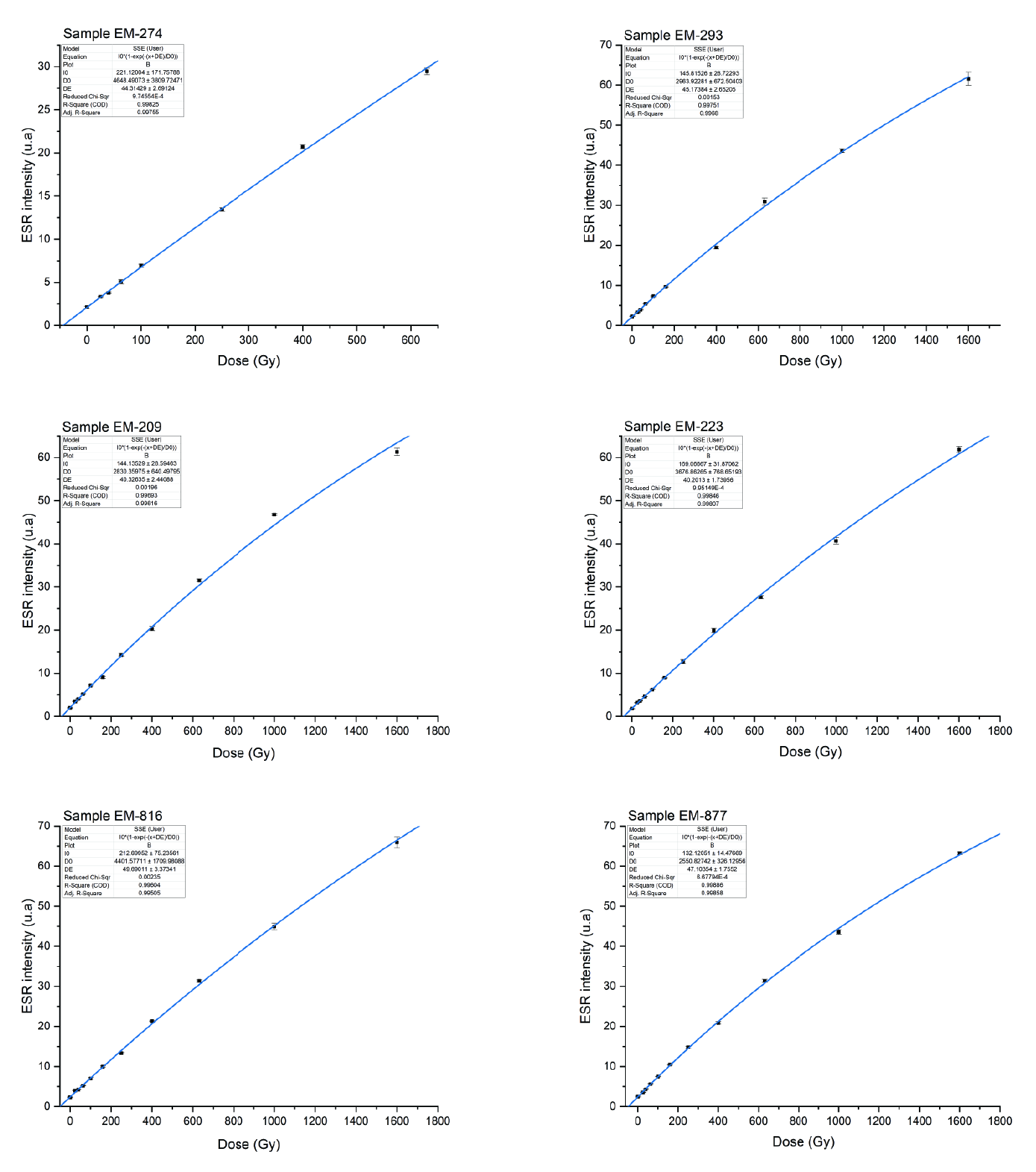

Supplement: S3 Fig — Fitting details are indicated on each DRC. (TIF) [file pone.0261282.s012.tif]

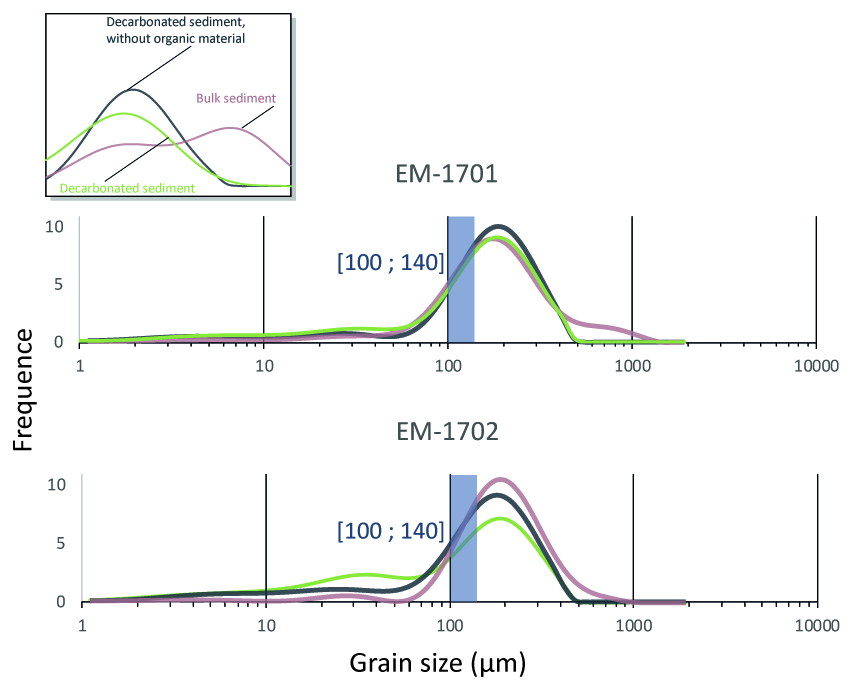

Supplement: S4 Fig — (TIF) [file pone.0261282.s013.tif]

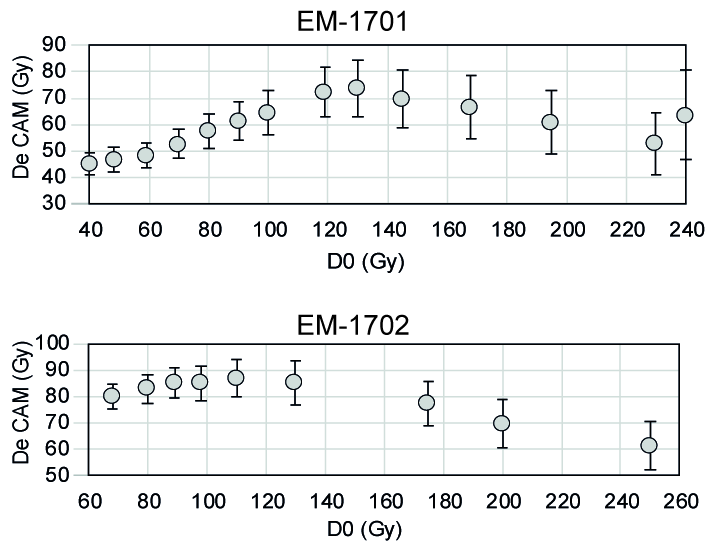

Supplement: S5 Fig — (TIF) [file pone.0261282.s014.tif]

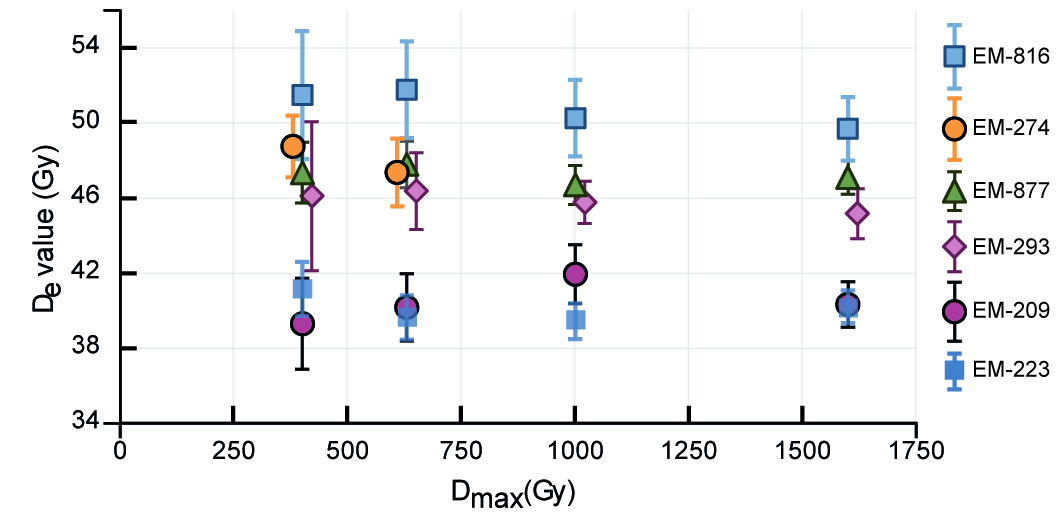

Supplement: S6 Fig — 1 σ errors are displayed. (TIF) [file pone.0261282.s015.tif]

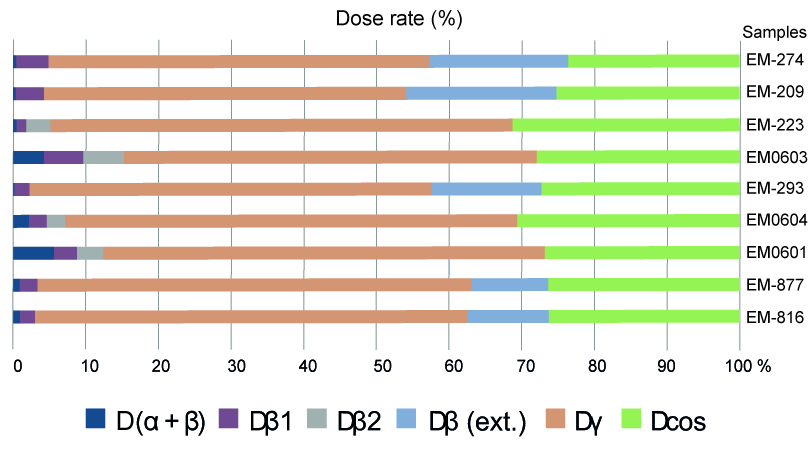

Supplement: S7 Fig — Key: Internal = dose-rate α + β contribution from the enamel, β1 = beta contribution from the dentine, β2 = beta contribution from cement or β (ext.) = beta contribution from sediment. (TIF) [file pone.0261282.s016.tif]

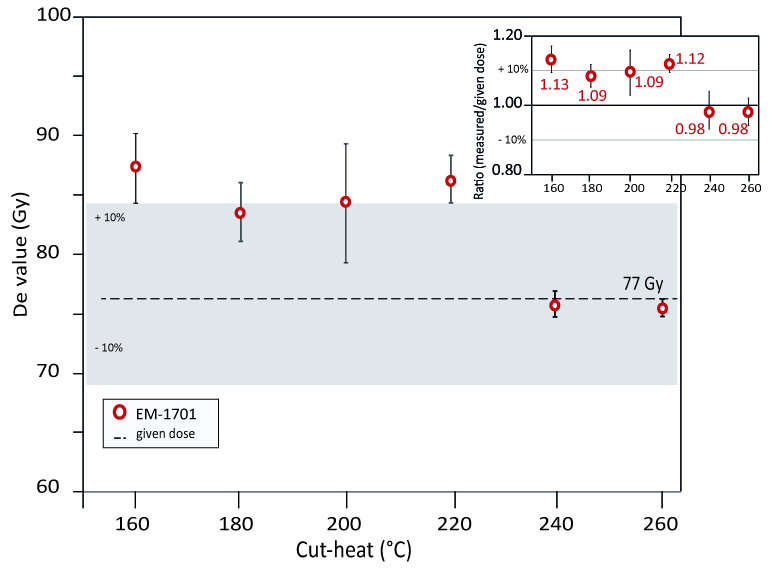

Supplement: S8 Fig — Each point corresponds to an average of 3 aliquots. (TIF) [file pone.0261282.s017.tif]

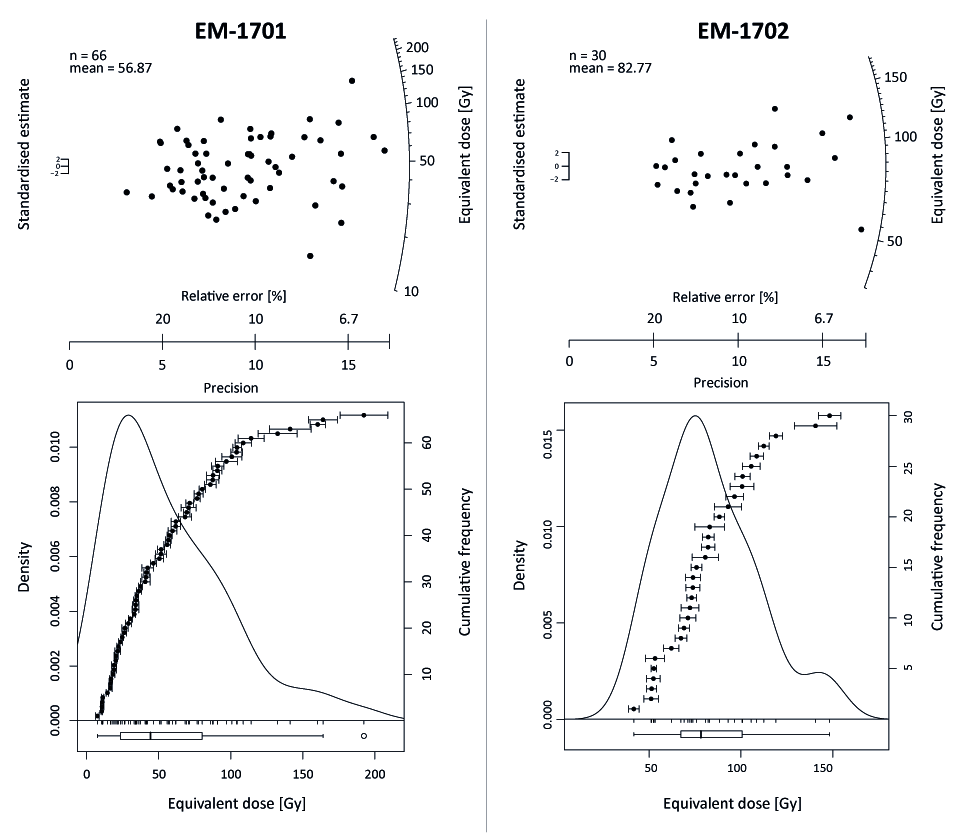

Supplement: S9 Fig — (TIF) [file pone.0261282.s018.tif]

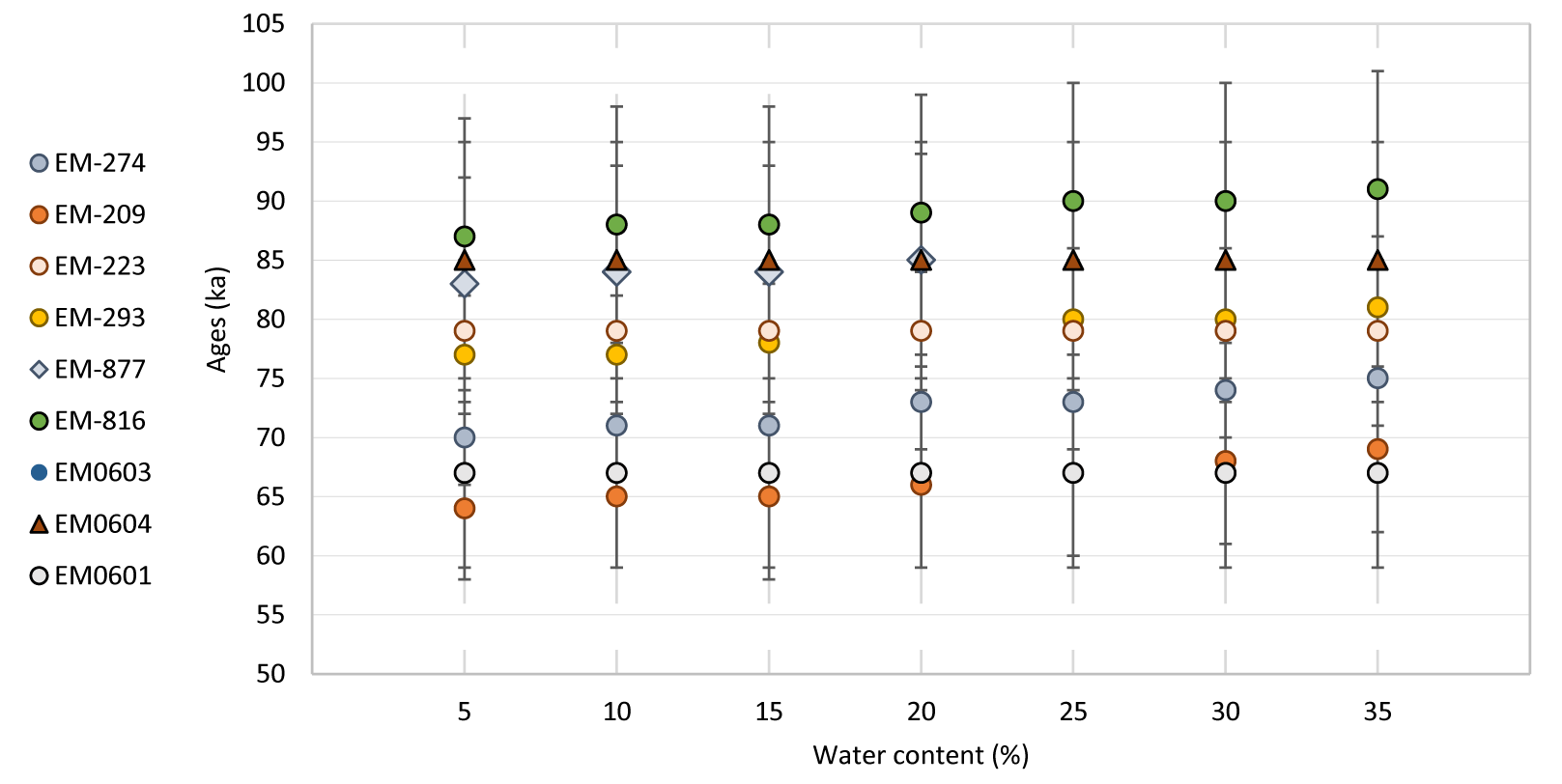

Supplement: S10 Fig — (TIF) [file pone.0261282.s019.tif]

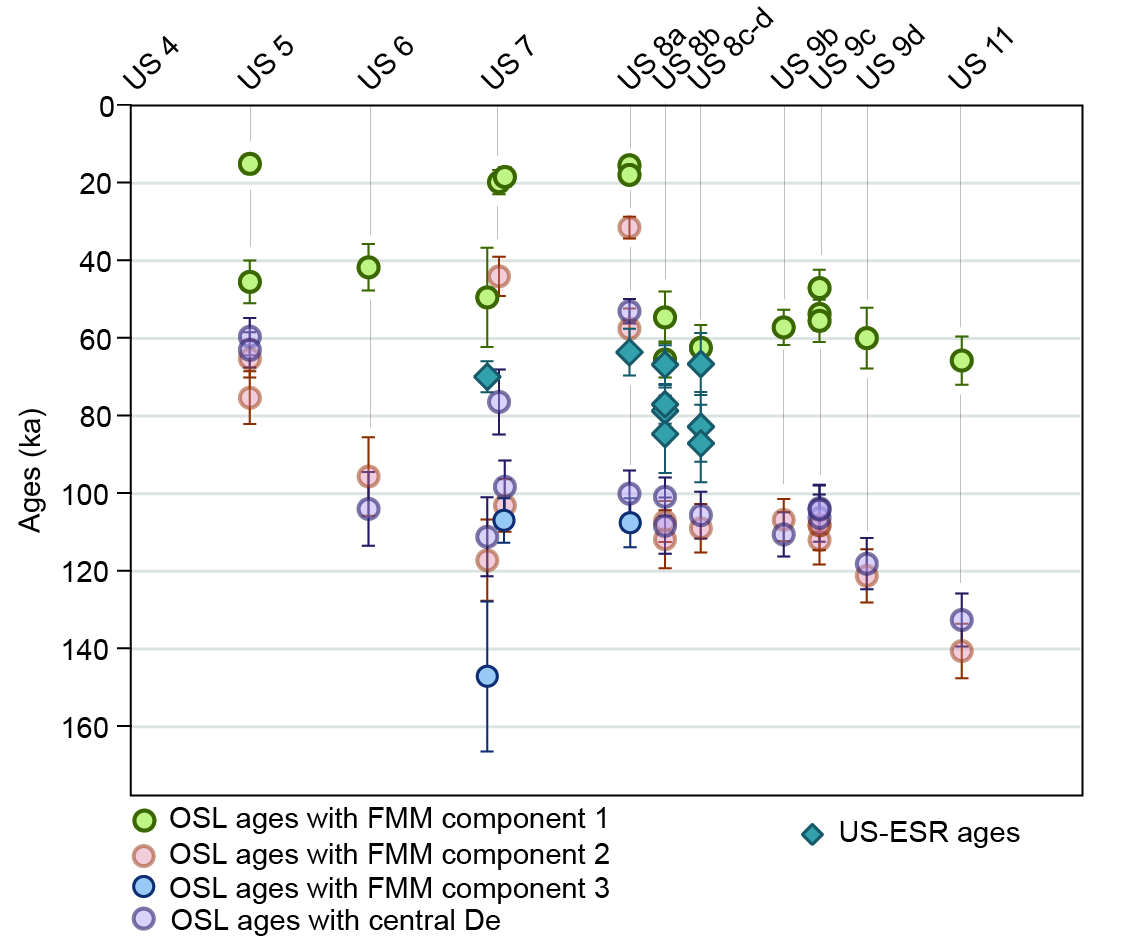

Supplement: S11 Fig — (TIF) [file pone.0261282.s020.tif]

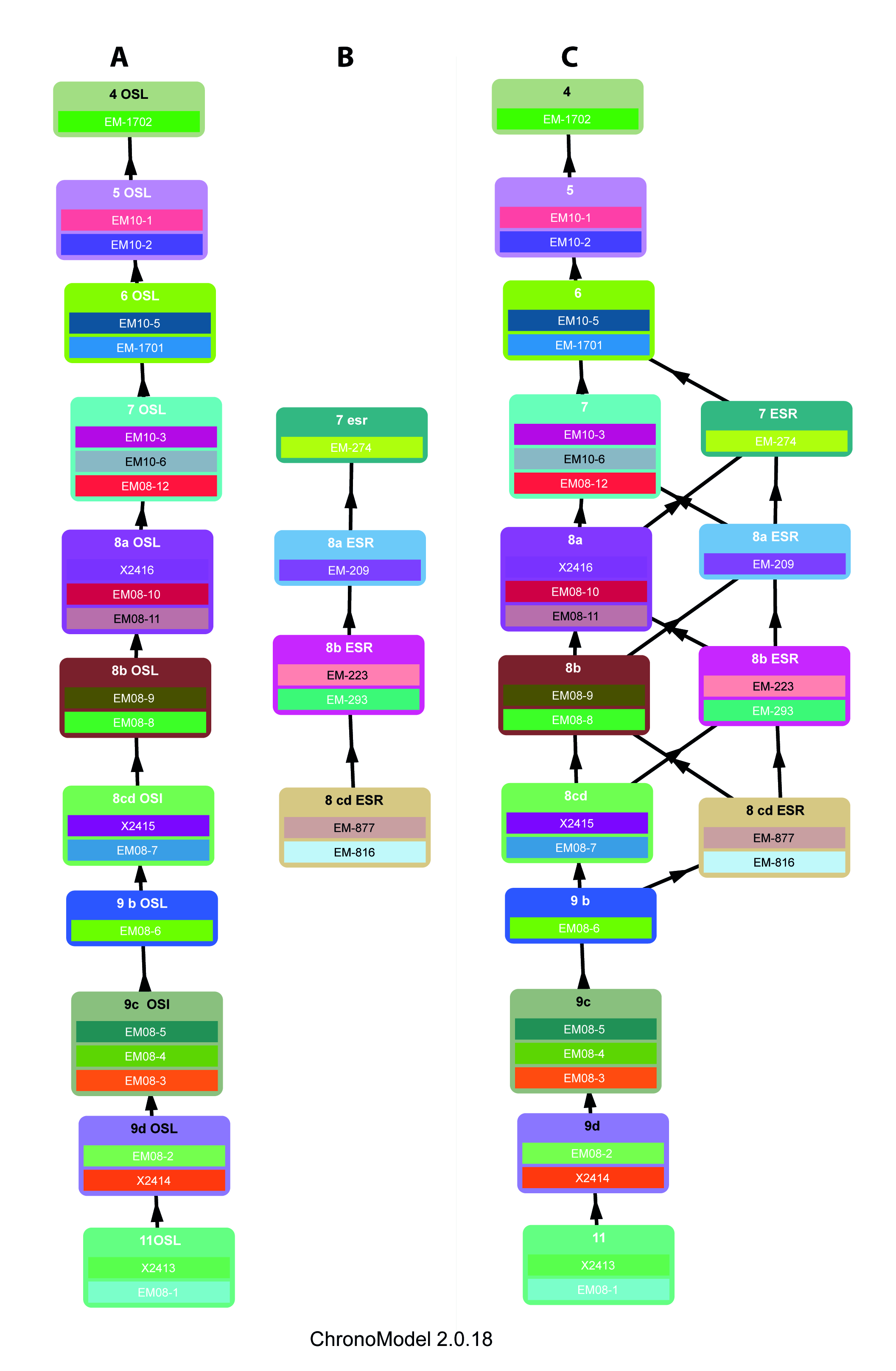

Supplement: S12 Fig — The arrow indicates stratigraphic constraints. The position of the OSL and US-ESR ages are given in the Table 1. A: OSL ages; B: combined US-ESR ages; C: global with all ages. (TIF) [file pone.0261282.s021.tif]
