# Supplementary material for: An improved chronology for the Middle Stone Age at El Mnasra cave, Morocco
Source: PLoS One. 2022 Feb 11;17(2):e0261282. doi: 10.1371/journal.pone.0261282 (PMC8836329; doi:10.1371/journal.pone.0261282)
Supplement: S1 File — (DOCX) [file pone.0261282.s022.docx]

**An improved chronology for the Middle Stone Age at El Mnasra cave, Morocco**

Eslem Ben Arous^1a, b, c^, Anne Philippe^d^ , Qingfeng Shao^e^, Olivier Tombret^b,f^, Norbert Mercier^g^, Maïlys Richard^h,g^, Daniel Richter^i^, Arnaud Lenoble^j^, Emmanuelle Stoetzel, Mohamed Abdeljalil El Hajraoui^k^, Roland Nespoulet^b^, Christophe Falguères^b^

Corresponding author: [ben-arous@shh.mpg.de](mailto:ben-arous@shh.mpg.de)

**Supplementary material**

S1 Text “Lithostratigraphic description and interpretation”

We describe the main geological sedimentation events in light of this new detailed stratigraphic reading. The sedimentary succession in El Mnasra cave shows that two main phases of sedimentation have taken place. The first one is during the shaping of the Ouljian cliff in which the cavity is dug during the high sea level of MIS 5e. Some beach deposits were directly trapped in the cavity (US 11), but the plateau above the Ouljian cliff could also host wind formations secondarily redistributed by runoff from the cave. The second period is the high sea level of the MIS 5c period, during which the sandy stock of the continental shelf is remobilised to form wind accumulations on the current coastline, traduced by the dune barrier giving the Oulja its current configuration [1].

First, a massive carbonate-rich dune was formed and then lithified, without sorting with shell debris. Dunes were trapped, from storms, in a cavity in the sharp cliff indicated that the coastline was probably close to the cave's entry. Sediments description could be indicated that the US 11 was MIS 5e and valid as a typical sequence for the ''Soltanian’’, as already proposed for Dar es Sotlan 1 sequence [2].

Sediment deposition in US 10 to 8 by runoff, leading firstly to regularisation of the pre-existing topography by sedimentation within puddles (US 10), then by progradation of a range of concentrated runoff or distal facies (US 9), which may include episodes of excavation/channel filling (e.g. US 9e). Sediments with an anthropogenic origin, such as charcoals, ashes, rubbed sediment, are showing human occupations of the site are present as early as US 9d. Although residual lenses of combustion activity represent them, these elements are dispersed. Thus, the possibility of preservation of archaeological occupations is relatively poor in the lower deposits of the US 9, giving rise to an anthropogenic fraction dispersed in natural sediment structured by runoff (US 9b and US 9d). However, US 9c, mainly formed of anthropogenic sediment, is an exception. On the contrary, preservation of the anthropogenic facies increases and becomes better in the US 9a, 8d and 8a.

Deposits of the US 10 to 8 represent the product of remobilisation by concentrated runoff from a coastal sedimentary stock mainly represented by aeolianites in a secondary position. Critically, the new sedimentary reading showing the geometry of the deposits indicates that this sedimentary stock is remobilised from the bottom of the cavity. It was not possible at this stage to determine whether the strata formed reflect new sedimentary entries into the cavity via the oculus or the reworking of a stock already accumulated in the deep parts of the cave. This point is particularly significant as it could have implications in grain bleaching and may generate heterogeneity in grain bleaching and apparent ageing of ages which could pose difficulties for luminescence approaches.

This reworking of aeolianite as the main sedimentary source is fading in US 7 and US 6, which consequently present a dominant anthropogenic fraction visible on section and attested during the excavation (ashes on the US 7, massive deposits with archaeological materials on the US 6). The presence of marine shells, forming well-delimited lenses and the fraction of carbonated sands still present in US 6 suggests a nearby coastline, highly probably during Marine Isotopic Stage (MIS) 5a.

In contrast, US 4 and 5 are characterised by a very clear decrease in grain size and a reddish colour. These two characteristics translate a continentalisation of the sedimentary stock by decreasing the sea level. Identical aeolian facies features were described regionally [3], and their construction should be placed during an arid period. In detail, the organic arenosols interspersed within US 5 suggest vegetation presence and a semi-arid environment, whereas their absence within US 4 could traduce desertification of the landscape. According to this lithostratigraphic data, US 5 and/or US 4 could be associated with MIS 4/3 or MIS 2.

US 3 results in very important bioturbation reworking the deposits and occasionally affects US 4, 5 and 6. This bioturbation reflects the occupation of the cavity by burrowing animals (e.g. Honey badgers), possibly at the end of the arid period and before the cavity was occupied during the Neolithic period (US 2).

S2 Text “Combined US-ESR dating”

- **Samples preparation and equipment**

ESR samples were prepared following the standard ESR dating protocol [4,5] and analysed at the Geochronology laboratory (MNHN, Paris). Dental tissues (enamel, dentine, cement) were separated mechanically. Enamel fragments were cleaned using a dental drill to remove at least 50 µm of enamel thickness from each side corresponding to the volume irradiated by alpha particles coming from other tissues. The clean enamel was then ground and sieved. Homogeneous enamel powder (100-200 µm in size) was split into 11 aliquots (each ~50 mg) and irradiated at increasing doses (0, 25, 40, 63, 100, 160, 250, 400, 630, 1000 and 1600 Gy) using a ^137^Cs γ-ray source at Centro Nacional de Investigación de La Evolución Humana (Gammacell, CENIEH, Burgos, Spain) with a constant dose-rate of 6.543 Gy/min. For sample EM-274, the small amount of enamel available was sufficient for only 9 aliquots (0, 25, 40, 63, 100, 160, 250, 400, 630 Gy).

- **ESR dose evaluation**

The evaluation of the equivalent dose is based on the multiple aliquot additive dose (MAAD) method. ESR measurements were carried out using a Bruker^©^ EMX X-band spectrometer at 18°C with the following measurement parameters: microwave power of 1.013 mW, 1024 points resolution, 9.80 GHz microwave frequency, 100 kHz modulation frequency, 1 G modulation amplitude, 20.48 ms conversion time and 5.1 ms time constant. Each aliquot was scanned 5 times for a total scanning time of 104.86 ms. The ESR measurements were repeated 4 times for each aliquot over different days to check the dispersion of the D_e_ values. A total of 240 ESR measurements were obtained in this study. ESR intensities were extracted from the asymmetric ESR signal between Top 1 and Base 2 (T1-B2) [6] using the Bruker WINEPR System software and normalised by the weight of each aliquot. Average ESR intensities from the repeated measurements were used to determine the equivalent dose (D_e_). Dose response curves (DRCs) were built from the measurements data set: D_e_ values were obtained by fitting a single saturated exponential (SSE) function to experimental data points [7]. SSE function is recommended for samples for which D_e_ are comprising between 10 and 100 Gy [8]. Data was fitted in Origin Pro (OriginLab Corporation, Northampton, USA) and weighted by the inverse of the squared ESR intensity (1/I^2^) [9]. All ESR dose response curves (DRC) with detailed fitting results are provided in S3 Fig.

**S3 Fig. ESR dose response curves (DRCs) computed by Origin Pro 8 software using Single Saturating Exponential (SSE) function.** Fitting details are indicated on each DRC.

- **Dosimetry**

Dosimetry in ESR dating of tooth enamel consists of the internal α (enamel) and β-dose-rates (enamel, dentine and cement), and external components from β- and γ-radiation of radioelements from the sediments containing the teeth, plus a cosmic dose. To calculate α- and β-dose-rates from dental tissues, U-series analyses were carried out using a ThermoScientific© Neptune Multi-Collector Inductively Coupled Plasma Mass Spectrometer (MC-ICPMS) in the Isotopic Mass Spectrometer Lab at Naǌing Normal University (China). 30-50 mg enamel and 2-5 mg cement/dentine were used for MC-ICPMS analysis following the method described by Shao et al. [10]. Uranium and thorium fractions were separated and purified using UTEVA® resin columns [11]. They were dissolved in a mixture of 0.5 N HNO_3_ and 0.01 N HF. Isotopic ratios ^234^U/^238^U, ^230^Th/^234^U, ^230^Th/^232^Th and radioelements contents were measured following Shao et al. [12]. Uranium isotopic ratios of standard uraninite HU-1 were measured every two samples to monitor instrumental stability [12]. Mass fractionation was corrected by comparing measured ^238^U/^235^U ratios to 137.82 value for natural samples with an exponential law [13]. As the individual γ-dosimetry for each sample is unknown, the external γ-dose-rates for each stratigraphic unit was assessed in situ with a γ-probe and with α-Al_2_O_3_:C dosimeter. Average γ-dose-rate per US was calculated [14,15].

Gamma-dose-rates *in situ* were measured using a portable γ-ray NaI-spectrometer (Inspector 1000 Canberra^©^) employing the threshold technique [16]. Measurements were performed in nine locations, in the West-profile, within squares 10 to 11 (Fig 5). Gamma-dose-rate was assessed *in situ* also by burying twelve α-Al_2_O_3_:C single crystal dosimeters for an entire year in US 7 to US 8c/8d [17,18]. Dosimeters were placed 30 cm deep into the sediments at appropriate locations for 4π dosimetry. The α-Al_2_O_3_:C passive dosimeters are shielded for α- and β-radiation and therefore measure the γ- and cosmic-dose-rates. In contrast to the instant results measured by portable γ-spectrometry, changes in γ-dose-rates caused by seasonal-variability of moisture in the sediment are taken into account with α-Al_2_O_3_:C passive dosimetry.

Analysis followed the protocol described in Richter et al. [19], where a Cs-137 irradiation individually calibrated the OSL response of 4-5 individual crystals within each dosimeter at the primary standards dosimetry laboratory (PSDL) of the Physikalisch Technische Bundesanstalt (PTB, Braunschweig, Germany).

External β-dose-rate was determined from U, Th, and K content of eight sediments sampled on the West-East profile within squares 10 to 11 (Fig 4), located at a distance of about 0.60m to 3.50-4.0m from the dated teeth samples. A ninth sediment was found in association with tooth EM-223. For this purpose, additional γ-spectroscopy measurements were performed with a high-resolution low-background Ortec® HpGe detector at the MNHN (Paris). About 100 g of sediment samples were sealed in plastic boxes and stored for three weeks to ensure radon equilibrium before conducting gamma spectrometry measurements. Dose-rate conversion factors are from Guérin et al. [20].

S3 Text “Optically Stimulated Luminescence dating”

- **Samples preparation and equipment**

Two sediment samples (EM-1701, EM-1702) were analysed using the OSL-facilities at the IRAMAT-CRP2A (Bordeaux Montaigne University, Pessac, France). Sediment at the extremities of the tubes was removed to avoid contamination from grains exposed to daylight during sampling. Removal of the sediment was performed in a dark room suitable for luminescence samples. Approximately 80-90 g of sediment per sample was sieved with water to isolate sand-sized grains for equivalent dose determination and dried at 50° C for 48 hours. Quartz grains in the size-fraction of 100-140 µm (S4 Fig) were chosen for their abundance and suitability for both multi-grain and single-grain SAR (Single Aliquot Regenerative) measurements. Sediment EM-1702 contained a higher carbonate fraction than sediment EM-1701. The sediments were prepared according to the standard protocol of IRAMAT-CRP2A: fractions were treated with an HCl solution (10%, 24h) to remove carbonates then with H_2_O_2_ (72h) to breakdown organic matter. Samples were rinsed, dried and dissolved in a solution of 10% HNO_3_ and 31% H_2_SiF_6_ to remove feldspars. Samples were rinsed with distilled water and HCl (10%, 24h) and dried.

**S4 Fig. Grain size on bulk sediment decarbonated sediment and decarbonated sediment without organic material.**

For quartz multi-grain measurements, a Freiberg Instruments lexsyg SMART automatic reader [63], equipped with a ^90^Sr/^90^Y β calibrated source with a dose rate of 0.154-0.152 ± 0.004 Gy/s. Green LEDs (525 nm, maximum power density of 50 mW/cm2) with a combination of UV filters (Schott BG-3, 3 mm in conjunction with a Delta BP365/50 EX) and an IR stimulation (850 ± 3 nm, 120 mW/cm²) were used. The OSL signal was detected by a photomultiplier tube (PMT) Hamamatsu H7360-02.

For quartz single-grain measurements, a Risø TL/OSL DA-20 reader, equipped with a 90Sr/90Y beta calibrated source (dose rate of 0.135 ± 0.004 Gy/s) was used. A PDM9107Q-AP-TTL-02 detected the OSL signal. Green laser stimulation (532 ± 25 nm, 50 mW/cm²) was applied with a 7.5 mm Hoya U-340 detection filter.

- **Equivalent dose determination**

Preliminary tests were carried out in the laboratory on multi-grain aliquots. An IR stimulation for 60s at 50 °C was applied before OSL measurement to eliminate potential contamination from remaining feldspar [21]. Grains were mounted on 9 mm base diameter stainless steel cups [22]. 18 aliquots of around 1 mm in diameter were measured for sample EM-1701. Dose recovery tests (DRT) and cut-heat plateau test were conducted (160–260°C, 20°C steps) for sample EM-1701. Three aliquots per temperature were measured after being artificially bleached in a Hönle SOL500 solar simulator for 1 h and for a given dose of 77 Gy. For measurements, the single aliquot regenerative (SAR) protocol [23,24] was applied with four regenerated doses, one repeat dose and one natural dose. The data were analysed with Analyst (Duller, 2015; v4.31).

For subsequent SG measurements, grains were loaded onto aluminium single grain discs, containing 100 holes (150 µm in diameter and 150 µm deep) drilled on a 10x10 rectangular grid. In order to ensure reliable estimation of the equivalent dose and using a single saturating exponential curve fitting, analysed grains were selected following the next criteria: (i) intersection with the dose response curve, (ii) deviation of recycling ratio within 10% from unity, (iii) a maximum recuperation of 5%, (iv) maximum error on the test dose of 10%, (v) Tn signal more than 3 sigmas above the background, (vi) grains with a D_0_ strictly smaller than D_e_ values.

S4 Text “US-ESR measurements”

- **Equivalent dose**

Equivalent dose values obtained range from around 40 to 50 Gy. Additional tests were made on the data set. An ESR sensitivity change test was performed: equivalent doses based on global mean ESR intensities used for final ages calculation were compared to individual equivalent doses derived from each independent series of measurements (S3 Table). Repeated ESR measurements (n=4) show relatively limited individual D_e_ variability (S3 Table) as the estimated dispersion of individual D_e_ and mean D_e_ per sample does not exceed 6 Gy on average (< 13%). A second test was performed to investigate the impact of the maximum irradiation dose (D_max_). D_max_ can affect D_e_ estimation as shown by Duval and Grün [8]: D_max_ should be comprised between 5 to 10* D_e_ values for samples with D_e_ <100 Gy. The impact of this parameter was also evaluated by fitting our data set by successively removing the last points of irradiation until reaching 10*D_e_, around 400 Gy. For sample EM-274, the small amount of enamel available did not allow sufficient irradiation and measurement of aliquots over 600 Gy. Re-calculated D_e_ shows a minor impact of the D_max_ on the current dataset (S6 Fig) as the difference between each D_e_ does not exceed 1-σ error (S4 Table) as shown for teeth dated in El Harhoura 2 site [5], a nearby cave.

**S3 Table. Comparison of the global mean equivalent doses to the individual equivalent doses derived from each independent series of measurements.** The dispersion estimated in % corresponds to the difference between the high and low D_e_ per serie.

|  |  |  | D_e_ (Gy) for each repeted measure time | | | | | | | |  |  |
| --- | --- | --- | --- | --- | --- | --- | --- | --- | --- | --- | --- | --- |
| Sample | D_e_ (Gy) | ± | Time 1 |  | Time 2 |  | Time 3 |  | Time 4 |  | D_e_ dispersion (%) | Adj. r² |
|  |  |  | D_e_ (Gy) | ± | D_e_ (Gy) | ± | D_e_ (Gy) | ± | D_e_ (Gy) | ± |  |  |
| EM-274 | **47.37** | **1.81** | 50.71 | 2.80 | 47.72 | 1.25 | 46.02 | 2.28 | 44.75 | 1.845 | 11.8 | 0.996 |
| EM-209 | **40.33** | **1.22** | 38.42 | 1.3 | 40.22 | 1.12 | 40.95 | 1.3 | 41.87 | 1.73 | 8.2 | 0.996 |
| EM-223 | **40.21** | **0.87** | 39.65 | 0.75 | 39.92 | 1.035 | 42.33 | 1.31 | 39.08 | 0.755 | 7.7 | 0.998 |
| EM-293 | **45.17** | **1.325** | 47.31 | 1.545 | 41.48 | 1.215 | 47.62 | 1.65 | 44.37 | 1.47 | 12.9 | 0.997 |
| EM-877 | **47.10** | **0.88** | 50.15 | 1.095 | 46.17 | 1.0175 | 44.09 | 0.81 | 48 | 1.23 | 13.7 | 0.999 |
| EM-816 | **49.69** | **1.69** | 51.13 | 1.74 | 51.59 | 1.7 | 47.42 | 1.755 | 48.8 | 1.915 | 5.7 | 0.995 |

**S6 Fig. D_e_ evolution as a function of D_max_ for all samples fitted with a SSE function.** 1 σ errors are displayed.

- **Dose-rate**

Uranium content and isotopic ratios are shown in S4 Table. While U contents are very low, ranging from 0.015 to 0.037 ppm in the enamel, they are comprised between 2.4 and 5.4 ppm in dentine. The ^234^U/^238^U ratios are homogeneous and systematically higher than 1. ^230^Th/^234^U ratios are smaller than 0.4 in all dental tissues. Therefore, there was no apparent uranium leaching precludes the determination of *p-values*. ^230^Th/^232^Th ratios are generally high in dentine and cement (up to 5750, S4 Table), whereas these ratios are smaller in enamel (between 15 and 80). These ratios are explained by a high ^230^Th content and a low ^232^Th content in dentine and cement. In enamel, ^230^Th/^232^Th ratio are lower. As uranium content in enamel is low (less than 37 ppb), ^230^Th/^232^Th ratios have a very low impact on US-ESR ages determination.

**S4 Table. U-series samples and data presented with 2σ error**.

| **Sample** | **Tissue** | **U-content (ppm)** | **±** | **^234^U/^238^U** | **±** | **^230^Th/^234^U** | **±** | **^230^Th/^232^Th** | **±** | **Enamel initial thickness (µm)** | **±** | **Removed thickness 1 (µm)** | **±** | **Removed thickness 2 (µm)** | **±** | **Type of analyses** |
| --- | --- | --- | --- | --- | --- | --- | --- | --- | --- | --- | --- | --- | --- | --- | --- | --- |
| EM-274 | D | 5.363 | 0.005 | 1.184 | 0.002 | 0.238 | 0.001 | 3729.068 | 465.739 |  |  |  |  |  |  | MC-ICPMS |
|  | E | 0.028 | 0.000 | 1.225 | 0.008 | 0.334 | 0.006 | 19.323 | 0.409 | 670 | 67 | 70 | 7 | 50 | 5 | MC-ICPMS |
| EM-209 | D | 4.684 | 0.004 | 1.176 | 0.002 | 0.258 | 0.001 | 3830.172 | 496.096 |  |  |  |  |  |  | MC-ICPMS |
|  | E | 0.030 | 0.000 | 1.203 | 0.007 | 0.224 | 0.006 | 59.649 | 3.379 | 690 | 69 | 74 | 7 | 54 | 5 | MC-ICPMS |
| EM-223 | D | 2.405 | 0.002 | 1.171 | 0.003 | 0.275 | 0.001 | 183.426 | 2.998 |  |  |  |  |  |  | MC-ICPMS |
|  | E | 0.021 | 0.000 | 1.243 | 0.009 | 0.297 | 0.007 | 34.190 | 1.594 | 1110 | 111 | 86 | 9 | 62 | 6 | MC-ICPMS |
|  | C | 3.910 | 0.003 | 1.190 | 0.002 | 0.321 | 0.001 | 253.503 | 2.283 |  |  |  |  |  |  | MC-ICPMS |
| EM0603* | D | *5.440* | *0.140* | *1.094* | *0.024* | *0.355* | *0.011* | *102.590* |  |  |  |  |  |  |  | α-spectrometry |
|  | E | *0.160* | *0.010* | *1.330* | *0.081* | *0.343* | *0.040* | *21.040* |  | 951 | 95 | 35 | 4 | 30 | 3 | α-spectrometry |
|  | C | *4.660* | *0.100* | *1.247* | *0.030* | *0.372* | *0.016* | *63.340* |  |  |  |  |  |  |  | α-spectrometry |
| EM-293 | D | 3.443 | 0.004 | 1.183 | 0.002 | 0.285 | 0.001 | 5750.007 | 1369.648 | 994 | 99 | 100 | 10 | 54 | 5 | MC-ICPMS |
|  | E | 0.015 | 0.000 | 1.229 | 0.010 | 0.332 | 0.009 | 14.430 | 0.490 |  |  |  |  |  |  | MC-ICPMS |
| EM0604* | D | *3.42* | *0.080* | *1.152* | *0.027* | *0.318* | *0.011* | *301.230* |  |  |  |  |  |  |  | α-spectrometry |
|  | E | *0.100* | *0.010* | *1.260* | *0.141* | *0.315* | *0.063* | *9.840* |  | 1160 | 116 | 30 | 3 | 30 | 3 | α-spectrometry |
|  | C | *3.620* | *0.070* | *1.185* | *0.021* | *0.300* | *0.012* | *131.100* |  |  |  |  |  |  |  | α-spectrometry |
| EM0601* | D | *3.560* | *0.100* | *1.147* | *0.030* | *0.347* | *0.013* | *91.660* |  |  |  |  |  |  |  | α-spectrometry |
|  | E | *0.190* | *0.010* | *1.999* | *0.063* | *0.358* | *0.038* | *58.410* |  | 1220 | 122 | 40 | 4 | 50 | 5 | α-spectrometry |
|  | C | *3.740* | *0.180* | *1.209* | *0.054* | *0.366* | *0.018* | *60.480* |  |  |  |  |  |  |  | α-spectrometry |
| EM-877 | D | 2.769 | 0.002 | 1.253 | 0.003 | 0.396 | 0.001 | 1015.337 | 38.696 | 1250 | 125 | 84 | 8 | 142 | 14 | MC-ICPMS |
|  | E | 0.027 | 0.000 | 1.284 | 0.009 | 0.399 | 0.007 | 46.476 | 1.883 |  |  |  |  |  |  | MC-ICPMS |
| EM-816 | D | 2.736 | 0.002 | 1.236 | 0.003 | 0.356 | 0.001 | 1819.422 | 164.009 | 1150 | 115 | 74 | 7 | 150 | 15 | MC-ICPMS |
|  | E | 0.037 | 0.000 | 1.290 | 0.007 | 0.374 | 0.007 | 78.412 | 3.452 |  |  |  |  |  |  | MC-ICPMS |

D=dentine; C=cement; E=enamel; side 1=dentine and side 2=sediment or cement. * : Data from [26] obtained previously with α-spectrometry.

Comparison of the radioelement's activities (S5 Table) from sediments samples shows a slight deficit of ^222^Rn for four samples (1805, 1807, 1808, 1809) of about 6.5 to 7.2 %. However, ^222^Rn activities are statistically consistent at 1-sigma with the ^238^U estimates and do not indicate significant disequilibrium. For each US, average ^40^K, ^238^U and ^232^Th contents were used to calculate β-dose-rates from sediment (S5 Table). The average concentrations range from 1.245 ± 0.029 to 1.422 ± 0.213 ppm for U, from 3.182 ± 0.536 to 3.516 ± 0.493 ppm for Th, and from 0.610 ± 0.110 to 0.739 ± 0.107 % for K. El Mnasra sediments mainly consist of sand, but also contain clay pockets, large carbonate contents, organic combustion areas and varying bone/shells density. This sediment diversity leads to a heterogeneous distribution of radioelements within the stratigraphical unit.

An average external γ-dose-rates was calculated considering the γ portable measurements acquired in this work, data from [26–28] (S6 Table) and the external γ-dose-rates given by Al_2_O_3_:C dosimeters (Table 3) : US 7, 8a, 8b and 8c/8d average γ-dose-rates were 356 ± 14 µGy/a, 316 ± 36 µGy/a, 325 ± 35 µGy/a and 340 ± 62 µGy/a, respectively.

**S5 Table. Radioelement activities (dpm/g) from HpGe γ-ray spectrometry of dry sediments collected at El Mnasra and converted in ppm.** The teeth corresponding to these measurements have been indicated. Sed-223: sediment directly associated to tooth EM-223 when it was taken from the collection. The averages calculated contents for these US are in blue and associated with standard deviation. A sedimentary description of the sediment and area sampled is given.

**S6 Table. El Mnasra dosimetry**.

|  | Al_2_O_3_:C dosimeters | | | | | | γ-spectrometry *in situ* | | | | | | | | |
| --- | --- | --- | --- | --- | --- | --- | --- | --- | --- | --- | --- | --- | --- | --- | --- |
|  | **Present work** | | | **Janati-Idrissi et al., 2012** | | | **Schwenninger et al., 2010** | | | **Jacobs et al., 2012** | | | **This work** | | |
|  | Squares H/G 9 to 11 | | | Squares H/G 9 to 11 | | | Squares H/G 7 to 8 | | | Squares H/G 7 to 10 | | | Squares H/G 9 to 11 | | |
| **US** | **ID-Lab** | **γ-dose-rate (µGy/a)*** | **±** | **ID-Lab** | **γ-dose-rate (µGy/a)*** | **±** | **ID-Lab** | **γ-dose-rate (µGy/a)** | **±** | **ID-Lab** | **γ-dose-rate (µGy/a)** | **±** | **ID-Lab** | **γ-dose-rate (µGy/a)** | **±** |
| 4 |  |  |  |  |  |  |  |  |  |  |  |  | EM-1702 | 462 | 23 |
| 5 |  |  |  |  |  |  |  |  |  | EM10-1 | 440 | 40 |  |  |  |
|  |  |  |  |  |  |  |  |  |  | EM10-2 | 490 | 30 |  |  |  |
| 6 |  |  |  |  |  |  |  |  |  | EM10-5 | 340 | 20 | EM-1701 | 324 | 16 |
| 7 | TL88 | 349 | 14 |  |  |  |  |  |  | EM10-6 | 370 | 30 | 2018-21 | 366 | 18 |
|  | TL89 | 339 | 16 |  |  |  |  |  |  | EM10-3 | 370 | 30 | 2018-22 | 361 | 18 |
|  |  |  |  |  |  |  |  |  |  | EM08-12 | 340 | 10 |  |  |  |
|  |  |  |  |  |  |  |  |  |  |  |  |  |  |  |  |
|  | **Average US 7 :** | **356** | **14** |  |  |  |  |  |  |  | | |  |  |  |
| 8a | TL148 | 265 | 32 |  |  |  | X2416 | 318 | 16 | EM08-11 | 320 | 10 | 2018-23 | 356 | 18 |
|  | TL149 | 270 | 27 |  |  |  |  |  |  | EM08-10 | 330 | 10 | 2018-24 | 351 | 18 |
|  |  |  |  |  |  |  |  |  |  |  |  |  |  |  |  |
|  | **Average US 8a :** | **316** | **36** |  |  |  |  |  |  |  | | |  |  |  |
| 8b | TL155 | 347 | 30 |  |  |  |  |  |  | EM08-9 | 360 | 10 | 2018-25 | 306 | 15 |
|  | TL156 | 293 | 24 |  |  |  |  |  |  | EM08-8 | 340 | 10 | 2018-26 | 359 | 18 |
|  | TL531 | 273 | 33 |  |  |  |  |  |  |  |  |  |  |  |  |
|  |  |  |  |  |  |  |  |  |  |  |  |  |  |  |  |
|  | **Average US 8b :** | **325** | **35** |  |  |  |  |  |  |  | | |  |  |  |
| 8c/8d | TL542 | 334 | 29 | EM0603 | 394 | 20 | X2415 | 353 | 18 | EM08-7 | 450 | 10 | 2018-27 | 355 | 18 |
|  | TL543 | 259 | 67 |  |  |  |  |  |  |  |  |  | 2018-28 | 356 | 18 |
|  | TL544 | 278 | 35 |  |  |  |  |  |  |  |  |  |  |  |  |
|  | TL545 | 250 | 43 |  |  |  |  |  |  |  |  |  |  |  |  |
|  | TL546 | 370 | 112 |  |  |  |  |  |  |  |  |  |  |  |  |
|  |  |  |  |  |  |  |  |  |  |  |  |  |  |  |  |
|  | **Average US 8c/8b :** | **340** | **62** |  |  |  |  |  |  |  | | |  |  |  |

Bold: averages γ-dose-rate associated error within one standard deviation used for ages calculation. *: cosmic dose-rates from Jacobs et al. [27] subtracted: 160 ± 20 µGy/a for US 6, 7, 8a, 8b and 150 ± 20 µGy/a for US 8c/8d.

These results show that the internal enamel dose is negligible with less than 7% of the annual dose (S7 Fig). The external and cosmic doses have a more considerable contribution, respectively around 75-80 % and up to 21%. Tooth EM-223 has the lowest annual dose (511 ± 40 μGy/a), which is 14% lower than the annual dose of tooth EM-293 from the same US (533 ± 40 μGy/a). This value can be explained by the presence of cement, which provides a β-dose-rate smaller than surrounding sediment.

**S7 Fig. Proportion of the different US-ESR dose-rate components.** Key: Internal = dose-rate α + β contribution from the enamel, β1 = beta contribution from the dentine, β2 = beta contribution from cement or β (ext.) = beta contribution from sediment.

S5 Text “Luminescence measurement”

The dose recovery test results for multi-grain measurements are shown in S8 Fig. A plateau for cut-heat temperatures of 240°C and 260°C with a dose recovery ratio close to unity were obtained for sample EM1701, indicates that we can measure a laboratory dose without major problems with our SAR protocol. A cut-heat temperature of 240 °C and a standard pre-heat temperature of 260 °C for 10s were thus used for subsequent measurements [29].

El Mnasra quartz samples thus met all laboratory-based criteria for a reliable estimate of the equivalent dose. 1000 single grains were measured: 30 and 66 D_e_ values for samples EM-1701 and EM-1702 respectively passed the rejection criteria-single saturating exponential curve fitting, and were used to calculate single-grain OSL ages. 6-13% of all measured single sand-sized quartz grains are suitable for OSL dating using the SAR protocol.

**S8 Fig. Cut-heat test combined with the DRT for the sample EM-1701 for a standard PH temperature at 260°C.** Each point corresponds to an average of 3 aliquots.

El Mnasra samples yield single-grain distributions with overdispersion (OD) of 30 and 72 % for samples EM-1702 and EM-1701, respectively. The OD of the sample EM1701 is significantly greater than the OD of the D_e_ distribution from El Mnasra samples dated by Jacobs et al. [27], between 33 and 40 %. Critically, both sediments' radial plot (S9 Fig) does not highlight clear grains delimited population, characteristic for well-bleached and unmixed samples. Informatively, we tested the FMM for 2-4 discrete dose components. Overdispersion values (σb) of 15, 20, 25, and 30% were employed as well as the Bayes Information Criterion (BIC) and the values of maximum log-likelihood (llik). We used them to check the possibility of assessing the minimum number of statistically supported D_e_ components for each sample [30,31]. The number of chosen components (k) is associated with the smallest BIC values [32]. There is no significant increase in maximum llik estimates. All FMM components and data are provided in S8 Table.

**S9 Fig.** **Radial plot and kernel density plot of equivalent dose distributions of single grains for samples EM-1701 and EM-1702, generated using RLumShiny package** [33]***.***

**S8 Table. Finite Mixture Model (FMM) details.**

**References**

1. Chahid D, Boudad L, Lenoble A, Hmaidi A El, Chakroun A, Jacobs Z. Nouvelles données morpho-stratigraphiques et géochronologiques sur le cordon littoral externe (SIM 5-c) de Rabat -Témara, Maroc. Géomorphologie Reli Process Environ. 2016;22: 253–264.

2. Barton RNE, Bouzouggar A, Collcutt SN, Schwenninger JL, Clark-Balzan L. OSL dating of the Aterian levels at Dar es-Soltan I (Rabat, Morocco) and implications for the dispersal of modern Homo sapiens. Quat Sci Rev. 2009;28: 1914–1931. doi:10.1016/j.quascirev.2009.03.010

3. Weisrock A, Adele B, Charif A, Tannouch-Bennani S. Dunes litorales et dunes continentales au Maroc Atlantique semi-arid (29^o^-30^o^ N) du Pléistocénes supérieur a l’actuel. Cuaternario y Geomorfol Rev la Soc Española Geomorfol y Asoc Española para el Estud del Cuaternario. 2002;16: 43–56. Available: https://dialnet.unirioja.es/servlet/articulo?codigo=3095766

4. Wagner GA, Krbetschek M, Degering D, Bahain J-J, Shao Q, Falgueres C, et al. Radiometric dating of the type-site for Homo heidelbergensis at Mauer, Germany. Proc Natl Acad Sci. 2010;107: 19726–19730. doi:10.1073/pnas.1012722107

5. Ben Arous E, Falguères C, Tombret O, El Hajraoui MA, Nespoulet R. Combined US-ESR dating of fossil teeth from El Harhoura 2 cave (Morocco): New data about the end of the MSA in Temara region. Quat Int. 2019;556: 88–95. doi:10.1016/j.quaint.2019.02.029

6. Grün R. Reproducibility measurements for ESR signal intensity and dose determination: High precision but doubtful accuracy. Radiation Measurements. 1998. doi:10.1016/S1350-4487(98)00014-6

7. Yokoyama Y, Falguères C, Quaegebeur JP. ESR dating of quartz from Quaternary sediments : first attempts. Nucl Tracks. 1985;10: 921–928.

8. Duval M, Grün R. Are published ESR dose assessments on fossil tooth enamel reliable? Quat Geochronol. 2016;31: 19–27. doi:10.1016/j.quageo.2015.09.007

9. Grün R, Brumby S. The assessment of errors in past radiation doses extrapolated from ESR/TL dose-response data. Radiat Meas. 1994;23: 307–315.

10. Shao Q, Bahain J-J, Falguères C, Peretto C, Arzarello M, Minelli A, et al. New ESR/U-series data for the early Middle Pleistocene site of Isernia la Pineta, Italy. Radiat Meas. 2011;46: 847–852. doi:10.1016/J.RADMEAS.2011.03.026

11. Horwitz EP, Dietz ML, Rhoads SM, Chiarizian R, Diamond H, Essling AM, et al. Separation and preconcentration of lead from acidic media by extraction chromatography. Anal Chim Acta. 1992;266: 25–37.

12. Shao QF, Li CH, Huang MJ, Liao ZB, Arps J, Huang CY, et al. Interactive programs of MC-ICPMS data processing for 230 Th/U geochronology. Quat Geochronol. 2019;51: 43–52. doi:10.1016/j.quageo.2019.01.004

13. Hiess J, Condon DJ, McLean N, Noble SR. 238U/235U systematics in terrestrial uranium-bearing minerals. Science (80- ). 2012;335: 1610–1614. doi:10.1126/science.1215507

14. Schwarcz HP. Current challenges to ESR dating. Quat Sci Rev. 1994;13: 601–605.

15. Mercier N, Valladas H, Valladas G, Reyss J-L. TL dates of burnt flints from Jelinek’s excavations at Tabun and their implications. J Archaeol Sci. 1995;22: 495–509.

16. Mercier N, Falguères C. Field gamma dose-rate measurement with a NaI(Tl) detector: re-evaluation of the “threshold” technique. Anc TL. 2007;25: 1–4.

17. Nespoulet R, El Hajraoui MA. Mission archéologique El Harhoura-Témara : Rapport d’activités. 2010.

18. Nespoulet R, El Hajraoui MA. Mission archéologique El Harhoura-Témara : Rapport d’activités. 2009.

19. Richter D, Dombrowski H, Neumaier S, Guibert P, Zink AC. Environmental gamma dosimetry with OSL of α-Al2O3:C for in situ sediment measurements. Radiat Prot Dosimetry. 2010;141: 27–35. doi:10.1093/rpd/ncq146

20. Guérin G, Mercier N, Adamiec G. Dose-rate conversion factor: update. Anc TL. 2011;29: 5–8.

21. Richard M, Mercier N, Charpentier V, Berger JF. OSL chronology of socio-ecological systems during the mid-Holocene in the eastern coast of the Sultanate of Oman (Arabian Peninsula). J Archaeol Sci Reports. 2020;33. doi:10.1016/j.jasrep.2020.102465

22. Richter D, Woda C, Dornich K. A new quartz for gamma-transfer calibration of radiation sources. Geochronometria. 2020.

23. Murray AS, Wintle AG. Luminescence dating of quartz using an improved single-aliquot regenerative-dose protocol. Radiat Meas. 2000;32: 57–73.

24. Murray AS, Wintle AG. The single aliquot regenerative dose protocol: Potential for improvements in reliability. Radiat Meas. 2003;37: 377–381. doi:10.1016/S1350-4487(03)00053-2

25. Duller G. The Analyst software package for luminescence data: overview and recent improvements. Anc TL. 2015;33: 35–42.

26. Janati-Idrissi N, Falguères C, Haddad M, Nespoulet R, Abdeljalil M, Hajraoui EL, et al. Datation par ESR-U/Th combinées de dents fossiles des grottes d’El Mnasra et D’ El Harhoura 2 , région de Rabat-Témara. Implications chronologiques sur le peuplement du Maroc Atlantique au Pléistocène Supérieur et son environnement. Quaternaire. 2012;23: 25–35.

27. Jacobs Z, Roberts RG, Nespoulet R, El Hajraoui MA, Debénath A. Single-grain OSL chronologies for Middle Palaeolithic deposits at El Mnasra and El Harhoura 2, Morocco: Implications for Late Pleistocene human-environment interactions along the Atlantic coast of northwest Africa. J Hum Evol. 2012;62: 377–394. doi:10.1016/j.jhevol.2011.12.001

28. Schwenninger J-L, Collcutt SN, Barton N, Bouzouggar A, Clark-Balzan L, El Hajraoui MA, et al. A New Luminescence Chronology for Aterian Cave Sites on the Atlantic Coast of Morocco. Oxbow Book. In: Garc, editor. South-Eastern Mediterranean Peoples Between 130, 000 and 10, 000 Years Ago. Oxbow Book. Oxbow Books; 2010. pp. 18–36.

29. Preusser F, Chithambo ML, Götte T, Martini M, Ramseyer K, Sendezera EJ, et al. Quartz as a natural luminescence dosimeter. Earth-Science Rev. 2009;97: 184–214. doi:10.1016/J.EARSCIREV.2009.09.006

30. Galbraith RF, Green PF. Estimating the component ages in a finite mixture. Int J Radiat Appl Instrumentation Nucl Tracks Radiat Meas Part D. 1990;17: 197–206. doi:10.1016/1359-0189(90)90035-V

31. Roberts RG, Galbraith RF, Yoshida H, Laslett GM, Olley JM. Distinguishing dose populations in sediment mixtures: A test of single-grain optical dating procedures using mixtures of laboratory-dosed quartz. Radiat Meas. 2000;32: 459–465. doi:10.1016/S1350-4487(00)00104-9

32. Galbraith RF, Roberts RG. Statistical aspects of equivalent dose and error calculation and display in OSL dating: An overview and some recommendations. Quat Geochronol. 2012;11: 1–27. doi:10.1016/j.quageo.2012.04.020

33. Burow C, Kreutzer S, Dietze M, Fuchs M, Fischer M, Schmidt C. RLumShiny - A graphical user interface for the R Package “Luminescence.” Anc TL. 2016;34: 22–32.
